# *Burkholderia* Bacteria Produce Multiple Potentially Novel Molecules that Inhibit Carbapenem-Resistant Gram-Negative Bacterial Pathogens

**DOI:** 10.3390/antibiotics10020147

**Published:** 2021-02-02

**Authors:** Eliza Depoorter, Evelien De Canck, Tom Coenye, Peter Vandamme

**Affiliations:** 1 Laboratory of Microbiology, Department of Biochemistry and Microbiology, Ghent University, 9000 Ghent, Belgium; eliza.depoorter@ugent.be (E.D.); evelien.decanck@ugent.be (E.D.C.); 2 Laboratory of Pharmaceutical Microbiology, Department of Pharmaceutical Analysis, Ghent University, 9000 Ghent, Belgium; tom.coenye@ugent.be

**Keywords:** *Burkholderia*, natural products, antimicrobial activity, ESKAPE pathogens, novel molecules

## Abstract

Antimicrobial resistance in Gram-negative pathogens represents a global threat to human health. This study determines the antimicrobial potential of a taxonomically and geographically diverse collection of 263 *Burkholderia* (sensu lato) isolates and applies natural product dereplication strategies to identify potentially novel molecules. Antimicrobial activity is almost exclusively present in *Burkholderia* sensu stricto bacteria and rarely observed in the novel genera *Paraburkholderia*, *Caballeronia*, *Robbsia*, *Trinickia*, and *Mycetohabitans*. Fourteen isolates show a unique spectrum of antimicrobial activity and inhibited carbapenem-resistant Gram-negative bacterial pathogens. Dereplication of the molecules present in crude spent agar extracts identifies 42 specialized metabolites, 19 of which represented potentially novel molecules. The known identified *Burkholderia* metabolites include toxoflavin, reumycin, pyrrolnitrin, enacyloxin, bactobolin, cepacidin, ditropolonyl sulfide, and antibiotics BN-227-F and SF 2420B, as well as the siderophores ornibactin, pyochelin, and cepabactin. Following semipreparative fractionation and activity testing, a total of five potentially novel molecules are detected in active fractions. Given the molecular formula and UV spectrum, two of those putative novel molecules are likely related to bactobolins, and another is likely related to enacyloxins. The results from this study confirm and extend the observation that *Burkholderia* bacteria present exciting opportunities for the discovery of potentially novel bioactive molecules.

## 1. Introduction

Antimicrobial resistance has become one of the most important threats to global health, causing at least 700,000 deaths worldwide each year [1]. Of particular concern are a group of microorganisms consisting of *Enterococcus faecium*, *Staphylococcus aureus*, *Klebsiella pneumoniae*, *Acinetobacter baumannii*, *Pseudomonas aeruginosa*, and *Enterobacter* spp., acronymically called the ‘ESKAPE’ pathogens [2]. Drug discovery efforts commonly suffer from low success rates, due to a lack of chemical diversity in the synthetic compound libraries. Such synthetic compounds are indeed much less likely to become antimicrobial drugs compared to microbial metabolites [3]. Bacterial natural products represent an important source of bioactive compounds with applications in agriculture and medicine [4]. Since the majority of currently known antibiotics are produced by actinomycetes, these organisms have been the main focus of drug discovery programs [5]. However, actinomycetes are not the only organisms with considerable genomic potential for specialized metabolite production, since recent bioinformatics analyses showed that specialized metabolite biosynthetic gene clusters are also widespread in the genomes of *Burkholderia* bacteria [6,7,8].

Bacteria belonging to the genus *Burkholderia* are well-known for their versatile metabolism and diverse lifestyles [9,10]. The heterogeneity of this genus has led to several taxonomical revisions [11,12,13,14], and therefore, the term *Burkholderia* is used below to indicate *Burkholderia* sensu lato. These organisms are frequently isolated from terrestrial and aquatic ecosystems, and can occur both free-living or in close association with fungi, protozoans, plants, animals, and humans [15,16,17,18,19]. Such interactions can either be beneficial or harmful to the eukaryotic host. For example, members of the *Burkholderia cepacia* complex (Bcc), a group of at least 22 closely related species, act as opportunistic pathogens infecting immunocompromised individuals, such as persons with cystic fibrosis (CF) [20,21]. Similarly, plant-pathogenic species, such as *Burkholderia gladioli*, *Burkholderia glumae*, and *Burkholderia plantarii*, affect economically important crops, such as rice, by producing several toxic compounds, including toxoflavin and tropolone [22,23].

In contrast, many *Burkholderia* bacteria have great potential for agricultural applications, due to their plant-growth-promoting and biopesticidal properties [24]. Several *Burkholderia* strains act as biological control agents by producing specialized metabolites, such as pyrrolnitrin, occidiofungin, and cepacins [25,26,27]. Although previous research was mainly focused on biocontrol through activity against plant-pathogenic fungi, there is a recent interest in employing *Burkholderia* bacteria in the search for novel antibiotics. Enacyloxins produced by *Burkholderia ambifaria* have activity against Gram-negative pathogens, such as *A. baumannii* [28]. Other examples include gladiolin, produced by *B. gladioli*, and thailandamides, produced by *Burkholderia thailandensis*, both of which have promising activity against *Mycobacterium tuberculosis* and *S. aureus*, respectively [29,30]. These recent discoveries, and the high number of ‘cryptic’ biosynthetic gene clusters found in the genomes of *Burkholderia* bacteria indicate that these organisms present a promising source for drug discovery [8]. The present study aimed to determine the antimicrobial potential of a diverse collection of *Burkholderia* sensu lato bacteria in search of novel antagonistic molecules.

## 2. Results

### 2.1. Exploring the Antimicrobial Potential of Burkholderia Isolates

A diverse collection of 263 *Burkholderia* isolates was selected from the BCCM/LMG Bacteria collection and our in-house strain collection based on isolation source, geographical origin, and taxonomic diversity (Table S1). This included the type strains of 97 validly named *Burkholderia* species, some of which have since been reclassified as members of the novel genera *Paraburkholderia*, *Caballeronia*, *Robbsia*, *Trinickia*, and *Mycetohabitans*. The majority of isolates (71%) originated from environmental sources, such as bulk soil, rhizosphere soil, plant material, including root nodules, freshwater, and sediment. The remaining isolates were obtained from clinical sources, including blood and CF sputum (Table 1).

An overlay assay [28] was used to search *Burkholderia* isolates with activity against *S. aureus* ATCC 29213, *A. baumannii* LMG 10520, and *Candida albicans* SC5314, as representatives of Gram-positive bacterial, Gram-negative bacterial and fungal pathogens, respectively. A total of 103 isolates inhibited at least one of the three pathogens tested, of which 97 belonged to *Burkholderia* sensu stricto (Table 1). These consisted of 70 Bcc isolates, 23 *B. gladioli* group bacteria, and four *Burkholderia pseudomallei* group bacteria. Only six isolates with antimicrobial activity belonged to the genera *Paraburkholderia* (*n* = 3) and *Trinickia* (*n* = 3), whereas no active isolates were observed for the genera *Caballeronia*, *Mycetohabitans*, and *Robbsia* (Table 1). No major differences in antimicrobial potential were observed between clinical isolates (43% of isolates active) and environmental isolates (38% of isolates active).

*Burkholderia arboris*, *Burkholderia contaminans*, and *Burkholderia pyrrocinia* strains were primarily active against *C. albicans* SC5314, whereas *B. glumae* and *B. plantarii* strains were especially active against *S. aureus* ATCC 29213. Overall, the proportion of isolates displaying activity against *S. aureus* ATCC 29213 (27%) and *C. albicans* SC5314 (29%) was very similar (Table 1). Activity against *A. baumannii* LMG 10520 was observed in 19 isolates (7%) belonging to *B. ambifaria*, *B. gladioli*, *B. cepacia*, *Burkholderia cenocepacia*, and eight unclassified Bcc isolates referred to as Other Bcc groups I and N [32]. To further examine this rather uncommon activity against Gram-negative bacterial pathogens, all 19 isolates with activity against *A. baumannii* LMG 10520 were selected for further analyses to determine the spectrum of activity. This set of isolates was further complemented with three isolates that inhibited *S. aureus* ATCC 29213 only, two isolates that inhibited *C. albicans* SC5314 only, and three isolates that inhibited both *S. aureus* ATCC 29213 and *C. albicans* SC5314.

### 2.2. Determining the Spectrum of Antimicrobial Activity of Burkholderia Isolates That Inhibit Gram-Negative Pathogens

To define the spectrum of antimicrobial activity, the resulting collection of 27 *Burkholderia* isolates was tested for activity against a panel of 34 human pathogens, consisting of ESKAPE pathogens, CDC-Urgent Threat pathogens (*Clostridioides difficile* and carbapenem-resistant Enterobacterales), and the fungal pathogens *Candida glabrata*, *Candida krusei* and *Aspergillus fumigatus* (Table S2). Per species, at least one strain with resistance to therapeutically relevant antibiotics or antifungals was tested.

Fourteen isolates inhibited carbapenem-resistant strains of *Enterobacter* spp., *Escherichia coli*, *Citrobacter freundii*, and *Morganella morganii* (Table 2). Nine of those also inhibited all three strains of *K. pneumoniae*, among which *B. cepacia* LMG 1222^T^, *B. cenocepacia* R-1474, and seven isolates identified as Other Bcc group I (Table 2). None of the *Burkholderia* isolates inhibited *P. aeruginosa* strains. Activity against Gram-positive pathogens was common, as 25 *Burkholderia* isolates inhibited all three strains of at least one Gram-positive pathogen and 14 of those *Burkholderia* isolates inhibited the growth of all *S. aureus*, *E. faecium*, and *C. difficile* strains tested (Table 2). *B. arboris* R-8833 was the only strain without activity against Gram-positive bacteria (Table 2). A total of 13 *Burkholderia* isolates inhibited all three strains of *A. fumigatus*, five of which also inhibited all three strains of both *C. glabrata* and *C. krusei*. These isolates with broad antifungal activity belonged to *B. ambifaria*, *B. arboris*, *B. gladioli*, and *Burkholderia vietnamiensis* (Table 2).

### 2.3. Extraction, Identification, and Dereplication of Burkholderia Specialized Metabolites

The results of the initial screen (Table 1) and the spectrum of activity (Table 2) were used to select 14 *Burkholderia* isolates for further analyses. These 14 isolates inhibited at least two out of three strains of at least two Gram-negative pathogens tested. Crude methanol extracts from spent Basal Salts Medium supplemented with glycerol (BSM-G) agar were subjected to liquid chromatography coupled to high-resolution mass spectrometry (LC-HRMS). A total of 42 metabolites were detected in the 14 crude extracts and included 23 previously characterized compounds present in the Chapman and Hall Dictionary of Natural Products (DNP) database (Table 3, Figure S1). The total number of metabolites detected in each crude extract varied from two (*B. glumae* R-8618) to 15 (Other Bcc I R-12632). Known molecules detected included enacyloxin, toxoflavin, reumycin, pyrrolnitrin, aminopyrrolnitrin, ornibactins, pyochelin, bactobolin A, cepacidin, ditropolonyl sulfide, cepabactin (=antibiotic BN-227), antibiotic BN-227-F, antibiotic SF 2420B, aerugine, aeruginoic acid, dihydroaeruginoic acid, and the signaling molecules cyclic guanosine monophosphate and differolide; the remaining 19 metabolites were not present in the DNP database, and thus, potentially represented novel molecules (Table 3, Figure S1).

### 2.4. Semipreparative Fractionation of Crude Extracts and Dereplication of Active Fractions

The crude extracts of eight *Burkholderia* strains that, together, produced 19 potentially novel metabolites, were subjected to semipreparative fractionation, and each fraction was tested for antimicrobial activity against *A. baumannii* LMG 10520 and *C. freundii* R-67508 (Table S2). The latter two pathogen strains were chosen based on the obtained inhibition spectra (Table 2), so that each of the eight *Burkholderia* isolates inhibited at least one of the two selected pathogens. Active fractions were analyzed through LC-HRMS, and the detected compounds were again identified through comparisons with the DNP database. 

**Figure 1:**
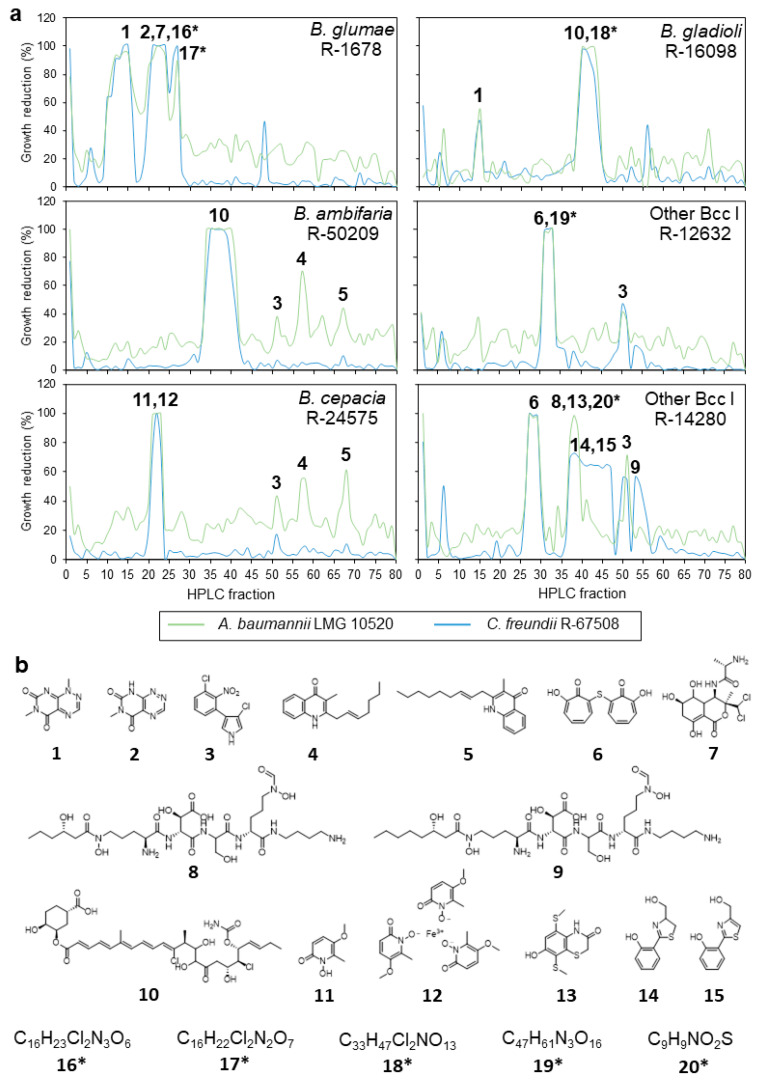
Antimicrobial activity and specialized metabolites detected in semipreparative fractions of six *Burkholderia* isolates. (**a**) Growth reduction observed per HPLC fraction. Numbers indicate specialized metabolites detected in active fractions and correspond with the chemical structures in panel b. (**b**) Chemical structures of compounds detected in active fractions. 1, reumycin; 2, toxoflavin; 3, pyrrolnitrin; 4, C_17_H_21_NO; 5, antibiotic SF 2420B; 6, ditropolonyl sulfide; 7, bactobolin A; 8, ornibactin C6; 9, ornibactin C8; 10, enacyloxin IIb; 11, cepabactin; 12, antibiotic BN-227-F; 13, C_10_H_11_NO_2_S_3_; 14, aerugine; 15, aeruginol; 16, C_16_H_23_Cl_2_N_3_O_6_; 17, C_16_H_22_Cl_2_N_2_O_7_; 18, C_33_H_47_Cl_2_NO_13_; 19, C_47_H_61_N_3_O_16_; 20, C_9_H_9_NO_2_S. Asterisks indicate putative novel compounds.

For each isolate, between two and seven active fractions were observed (Table 4), except for the strain *Burkholderia singularis* LMG 28155, for which none of the fractions showed antimicrobial activity (Figure S2). Although their spectrum of antimicrobial activity differed (Table 2), the results obtained for the two *B. gladioli* isolates (R-16098 and R-27098) proved very similar, both in terms of high-performance liquid chromatography (HPLC) profile and antibacterial activity of the fractions (Figures S2 and S3). Three molecules were detected in the active fractions of both *B. gladioli* isolates, among which reumycin, enacyloxin, and a putative novel compound with molecular formula C_33_H_47_Cl_2_NO_13_ (Table 3, Figure 1). Enacyloxin was also detected in an active fraction of *B. ambifaria* R-50209, as well as pyrrolnitrin and two quinolinone antibiotics (antibiotic SF 2420B and a known C_17_H_21_NO compound). Fractions containing the latter three molecules exhibited only moderate activity (40–65% growth reduction) against *A. baumannii* LMG 10520 and weak to no activity against *C. freundii* R-67508, and those molecules were also detected in active fractions of *B. cepacia* R-24575 (Table 4, Figure 1). A fourth fraction of the latter isolate had strong activity against both pathogens and consisted of a mixture of cepabactin and its ferric iron chelate BN-227-F (Figure 1).

Several highly active fractions were observed for *B. glumae* R-1678, containing reumycin, toxoflavin, bactobolin A, and two potentially novel compounds with molecular formulas C_16_H_23_Cl_2_N_3_O_6_ and C_16_H_22_Cl_2_N_2_O_7_. Only two active fractions were found for Other Bcc I R-12632, one containing pyrrolnitrin and a second fraction containing a mixture of ditropolonyl sulfide and a putative novel compound with molecular formula C_47_H_61_N_3_O_16_ (Table 4). The second Other Bcc I isolate R-14280 also produced pyrrolnitrin and ditropolonyl sulfide, and several additional active fractions contained mixtures of ornibactins C6 and C8, aerugine, aeruginol, C_10_H_11_NO_2_S_3_, and a putative novel compound with molecular formula C_9_H_9_NO_2_S (Table 4).

## 3. Discussion

To explore the antimicrobial potential of *Burkholderia* sensu lato bacteria, a taxonomically and geographically diverse collection of isolates was screened for antagonistic activity against *S. aureus* ATCC 29213, *A. baumannii* LMG 10520, and *C. albicans* SC5314. Of the 103 isolates demonstrating the antimicrobial activity, 97 belonged to *Burkholderia* sensu stricto [6] (Table 1). Although almost a third of the screened isolates belonged to *Burkholderia* species now reclassified into the novel genera *Paraburkholderia*, *Caballeronia*, *Robbsia*, *Trinickia*, and *Mycetohabitans* [11,12,13,14], antimicrobial activity was detected in only six out of 87 strains tested. This was the case for *Paraburkholderia phenazinium* LMG 2247^T^, a known producer of antimicrobial phenazine pigments [33], and *Paraburkholderia bryophila* LMG 23664^T^, for which antifungal activity has been reported previously [34] (Table 1). Out of six *Paraburkholderia terricola* isolates tested, LMG 20594^T^ showed activity against *S. aureus* ATCC 29213 (Table 1), which might be due to the production of siderophores [35].

Activity against *S. aureus* ATCC 29213 and *C. albicans* SC5314 was rather common and occurred in 37.5% and 39.7% of *Burkholderia* sensu stricto isolates, respectively, whereas only 7.2% of isolates showed activity against *A. baumannii* LMG 10520 (Table 1). Although antagonistic activity against *A. baumannii* has previously been reported in strains of *B. ambifaria* [28], isolates of several other *Burkholderia* species inhibited the growth of this important Gram-negative pathogen (Table 2). These included isolates of *B. cepacia*, *B. cenocepacia*, *B. gladioli*, *B. glumae*, *B. singularis*, and the unclassified Bcc species designated Other Bcc groups I and N, which are closely related to *B. cenocepacia* [32].

Because antimicrobial activity against Gram-negative bacterial pathogens is uncommon and the need for novel therapeutic options for this group is urgent, the 19 *Burkholderia* isolates that inhibited *A. baumannii* LMG 10520 and eight additional isolates were analyzed to determine the broader spectrum of antimicrobial activity. Almost all isolates showed activity against one or more Gram-positive pathogens, except for *B. arboris* R-8833, which exhibited broad antifungal activity (Table 2). Interestingly, the first reported producer of enacyloxin, *B. ambifaria* LMG 19182^T^, appeared as two colony types (t1 and t2), which each presented a distinct spectrum of activity. The t1 colony type appeared as matte, yellow-pigmented colonies when grown on Tryptone Soya Agar (TSA) and showed antagonistic activity against *C. albicans* SC5314 and all three strains of *C. glabrata*, *C. krusei*, and *A. fumigatus* each. The t2 colony type appeared as shiny, off-white colonies when grown on TSA, and no antifungal activity was detected for the t2 colony type (Table 2). Multilocus sequence typing [31,36] confirmed that both colony types presented an identical allelic profile corresponding to ST-77 (https://pubmlst.org/bcc/), thus, ruling out contamination. Since strain LMG 19182^T^ has been reported to produce several antifungal substances [27,28,37], it is possible that the regulation and expression of one or more of the associated biosynthetic gene clusters has been altered in the t2 colony type.

Several isolates inhibited carbapenem-resistant strains of Gram-negative pathogens, *Enterobacter* spp., *E. coli*, *C. freundii*, and *M. morganii*, and nine isolates also inhibited the growth of all three strains of *K. pneumoniae* (Table 2). None of the *Burkholderia* isolates inhibited *P. aeruginosa* strains, which confirmed earlier reports [28].

Fourteen *Burkholderia* isolates showed a unique spectrum of activity, including antibiotic activity against at least two Gram-negative pathogens (Table 2). For each of those isolates, a spent agar extract was prepared and analyzed through LC-HRMS to detect and identify the specialized metabolites produced. A total of 42 metabolites was detected, 23 of which represented known molecules (Table 3). This included several siderophores, such as ornibactins, pyochelin, cepabactin, and its ferric iron chelate antibiotic BN-227-F, all of which have been shown to exhibit antimicrobial activity [38,39,40,41]. These were exclusively detected in isolates of *B. cepacia*, *B. cenocepacia*, and related Other Bcc groups I and N (Table 3). Those eight isolates also produced ditropolonyl sulfide and the pyochelin-related compounds aerugine, aeruginoic acid, and dihydroaeruginoic acid. Whereas antibacterial activity has been reported for ditropolonyl sulfide [42], aerugine and related compounds have been described as antifungals [43,44]. Although the production of aeruginaldehyde, another aerugine-related compound, has been reported for several *Burkholderia* isolates [45,46], this molecule was not detected in any of the crude extracts (Table 3). Given the unstable nature of aeruginaldehyde, it is possible that this molecule was derivatized during the 4-day incubation period. Production of enacyloxin was restricted to isolates *of B. ambifaria* and *B. gladioli*, similar to what was observed in earlier studies [28,47]. On the other hand, toxoflavin and reumycin were only detected in *B. gladioli* and *B. glumae* isolates (Table 3). In addition, *B. glumae* R-1678 produced the antitumor antibiotic bactobolin A, which had previously only been isolated from *B. thailandensis* E264^T^ [48,49]. 

Out of the 42 detected metabolites, 19 did not result in a match in the DNP database, and were, therefore, considered potentially novel molecules (Table 3). The production of most of these molecules was strain-specific. Based on their molecular formula and UV spectrum (Figure S3), some putative novel molecules appeared closely related to existing *Burkholderia* natural products, such as ornibactins (C_26_H_48_N_9_O_14_, C_33_H_35_N_9_O_11_, C_35_H_39_N_9_O_11_, and C_37_H_43_N_9_O_11_), bactobolins (C_16_H_22_Cl_2_N_2_O_6_ and C_16_H_23_Cl_2_N_3_O_7_), and enacyloxins (C_33_H_47_Cl_2_NO_13_). 

In search of the potentially novel bioactive metabolites, crude agar extracts of eight *Burkholderia* isolates that, together, produced each of the 19 potentially novel molecules, were subjected to semipreparative fractionation, and the resulting fractions were tested for antibacterial activity against *A. baumannii* LMG 10520 and *C. freundii* R-67508. Twenty different specialized metabolites were found in active fractions, of which only five represented putative novel molecules (Table 4). Of these, only one (with the molecular formula C_33_H_47_Cl_2_NO_13_) was detected during the dereplication of the crude extracts (Table 3). Several fractions showed strong antibacterial activity towards both Gram-negative pathogens and contained toxoflavin, reumycin, enacyloxin, bactobolin, and two putative novel molecules likely related to bactobolins (Figure 1). Most of these metabolites have been extensively characterized and also exhibit antifungal, herbicidal, or cytotoxic activity [48,49,50,51]. Other fractions with strong antibacterial activity contained cepabactin, its ferric ion chelate antibiotic BN-227-F, and ditropolonyl sulfide (Table 4, Figure 1), all of which were discovered decades ago [38,39,42], but which have not been thoroughly characterized in terms of biosynthesis and regulation. Weak to moderate antibacterial activity was observed for fractions containing pyrrolnitrin, ornibactin, aerugine, and aeruginol (Table 4, Figure 1), which were observed in the crude extracts of multiple Bcc isolates (Table 3). Indeed, the production of the antifungal pyrrolnitrin and the siderophore family of ornibactins is a common feature observed in Bcc bacteria [52,53]. Although aerugine and related compounds have not been described as siderophores, they are structurally similar to pyochelin and share with it the iron-chelating compound salicylic acid as a precursor [54].

Although 19 potentially novel metabolites were detected in the crude extracts, only one of those (C_33_H_47_Cl_2_NO_13_) was detected in a fraction with antimicrobial activity (Table 4). A likely explanation is that most of these putative novel metabolites do not inhibit the two Gram-negative pathogens tested, but stability issues might also have been a confounding factor. On the other hand, four out of five putative novel metabolites detected in active fractions were not observed during the dereplication of the crude extracts (Table 3; Table 4). This phenomenon was also observed for known metabolites, such as reumycin (*B. gladioli* R-16098 and R-20794), pyrrolnitrin (*B. ambifaria* R-50209), and ditropolonyl sulfide (Other Bcc I R-12632). The fact that these molecules were not detected initially during the dereplication of the crude extracts is presumably due to a difference in chromatographic methods used to analyze the crude extracts and the fractionation. Semipreparative fractionation not only separates the mixture of compounds, thereby avoiding ionization interferences in the following LC-HRMS step, but it also results in fractions that are more concentrated than the crude extracts.

A total of five potentially novel metabolites were detected in active fractions of five *Burkholderia* isolates. *B. glumae* R-1678 produced two of those putative novel molecules, with molecular formulas C_16_H_22_Cl_2_N_2_O_6_ and C_16_H_23_Cl_2_N_3_O_7_, which likely represent new bactobolin derivatives (Table 4). The largest putative novel metabolite, with molecular formula C_47_H_61_N_3_O_16_, was produced by Other Bcc I R-12632 and was detected in a mixed fraction together with ditropolonyl sulfide (Table 4). Because inhibition of Gram-negative pathogens has been described previously for ditropolonyl sulfide [42], it remains unclear whether or not the C_47_H_61_N_3_O_16_ molecule is contributing to the observed activity. A similar situation was observed for one of the active fractions of Other Bcc I R-14280, which contained the putative novel molecule with molecular formula C_9_H_9_NO_2_S in a mixture with ornibactin C6 and a known molecule with molecular formula C_10_H_11_NO_2_S_3_ (Table 4). This mixed fraction had strong activity against *A. baumannii* LMG 10520 and moderate activity against *C. freundii* R-67508 (Figure 1, Table 4). Although bacteriostatic activity has been reported for ornibactin [40], it is unlikely that this siderophore is solely responsible for the observed inhibition. The known compound with molecular formula C_10_H_11_NO_2_S_3_ has been detected in cultures of *P. aeruginosa* transformed with a cryptic three-gene operon from *B. pseudomallei*, but antimicrobial activity was not reported [55]. Further fractionation and activity testing of this mixed fraction will be necessary to determine which of the three compounds contribute to the antimicrobial activity and to what extent. Finally, *B. gladioli* R-16098 and R-20794 both produced a putative novel molecule with molecular formula C_33_H_47_Cl_2_NO_13_, which is likely related to enacyloxins (Table 4, Figure S3). The fraction containing this putative novel metabolite, and the fractions containing enacyloxin all strongly inhibited the growth of both *A. baumannii* LMG 10520 and *C. freundii* R-67508.

## 4. Materials and Methods

### 4.1. Strains and Growth Media

A diverse collection of 263 *Burkholderia* isolates was selected from our in-house strain collection based on taxonomic diversity, isolation source, and geographical origin (Table S1). The producers of five known *Burkholderia* antimicrobials were included in this selection as positive controls: *B. ambifaria* LMG 19182^T^ (enacyloxin), *B. ambifaria* R-14455 (burkholdines), *B. pyrrocinia* LMG 14191^T^ (pyrrolnitrin), *B. pyrrocinia* LMG 21822 (xylocandins), and *Burkholderia diffusa* LMG 29043 (cepacins). All strains were routinely cultured on TSA (Oxoid, Basingstoke, UK) at 28 °C and maintained at −80 °C in Microbank™ vials. BSM-G agar [56] was used as the medium for antimicrobial production during the overlay assay. BSM-G had a pH of 7 and contained per liter: 3.24 g K_2_HPO_4_, 1.13 g NaH_2_PO_4_.2H_2_O, 2 g NH_4_Cl, 0.2 g MgSO_4_.7H_2_O, 0.1 g nitrilotriacetic acid, 4.7 g glycerol, 3 mg MnSO_4_.H_2_O, 3 mg ZnSO_4_.7H_2_O, 1 mg CoSO_4_.7H_2_O, 7.7 mg FeSO_4_.nH_2_O (*n* = 1.5) and 15 g agar.

A diverse set of pathogens was selected for antimicrobial activity screens and included representatives of Gram-positive and Gram-negative bacteria, yeasts, and filamentous fungi (Table S2). For the majority of pathogens, at least one strain with acquired resistance to antibiotics relevant in human medicine was selected. This included resistance to methicillin and vancomycin for Gram-positive bacteria, resistance to tetracyclines and carbapenems for Gram-negative bacteria, and azole resistance in the case of yeasts and fungi (Table S2).

### 4.2. Overlay Assay to Detect Antimicrobial Activity

*Burkholderia* isolates were tested for antimicrobial activity against a diverse panel of pathogens (Table S2) using an overlay method as described earlier [28]. *Burkholderia* isolates were grown on TSA at 28 °C for 48 h and were subcultivated twice prior to performing the overlay assay. For each strain, a dense suspension of approx. 1 × 10^8^ CFU/mL was prepared in sterile 0.85% saline and homogenized through vortexing. A 3 µL drop of this suspension was inoculated onto the center of a BSM-G agar plate, which was left to dry at room temperature. Plates were incubated at 28 °C for 4 days to allow the production of antimicrobials. Plates were then exposed to chloroform vapors for 5 min and left to air for at least 10 min to allow evaporation of the residual chloroform. Due to the variation in growth requirements of different pathogens, several variations of the overlay assay protocol were used.

For all bacterial pathogens except *C. difficile*, an overnight culture was grown at 37 °C in 10 mL Iso-Sensitest broth (ISB; Oxoid, UK). The resulting cultures were adjusted to an optical density at 590 nm (OD_590_) of 1. For Gram-negative strains, 100 µL of this suspension was inoculated into 100 mL soft agar (ISB with 1% agar) to obtain a suspension of approximately 1 × 10^5^ CFU/mL. For Gram-positive strains, 500 µL of the suspension was added into 100 mL of soft agar, corresponding to approximately 5 × 10^5^ CFU/mL. To stain growing bacteria and aid visualization of the inhibition zones, the soft agar was supplemented with triphenyl tetrazolium chloride (Sigma-Aldrich, St. Louis, MO, USA) to a final concentration of 0.02%. This protocol is referred to as ‘Protocol A’ (Table S2). For *C. difficile*, all manipulations were performed inside an anaerobic cabinet (Jacomex, Dagneux, France) in an atmosphere consisting of 80% N_2_, 10% H_2_, and 10% CO_2_. Overnight cultures of *C. difficile* were grown at 37 °C in Reinforced Clostridial Medium (RCM; Oxoid, UK), and 2 mL of this suspension was added to 100 mL of soft agar (RCM with 1% agar) to obtain approximately 5 × 10^5^ CFU/mL. This protocol is referred to as ‘Protocol B’ (Table S2). Overnight cultures of *Candida* spp. were grown at 37 °C in 10 mL Sabouraud Dextrose Broth (SDB; 40 g/L dextrose and 10 g/L yeast extract) and were adjusted to an OD_590_ of 1. Four mL of this suspension was added into 100 mL of soft agar (SDB with 1% agar) to obtain approximately 5 × 10^5^ CFU/mL. To stain growing yeasts and aid visualization of the inhibition zones, the soft agar was supplemented with thiazolyl blue tetrazolium bromide (Sigma-Aldrich, USA) to a final concentration of 0.5 mg/mL. This protocol is referred to as ‘Protocol C’ (Table S2). For *A. fumigatus*, cultures were grown on Sabouraud Dextrose Agar (SDB with 2% agar) at 28 °C for 7 days to obtain sporulating cultures. Spores were harvested in a 0.1% Tween20 solution and filtered using a 40 µm cell strainer (Corning, Corning, NY, USA) to remove residual mycelium. Four ml of this filtered spore suspension was added to 100 mL of soft agar (SDB with 1% agar) to obtain approximately 1 × 10^6^ spores/mL. This protocol is referred to as ‘Protocol D’ (Table S2).

Approximately 1.5 mL (12-well plates) or 15 mL (90 mm plates) of inoculated soft agar was gently poured over the *Burkholderia* growth, and allowed to set at room temperature for 1 h. Plates were incubated either at 37 °C for 24 h (protocols A and B), 37 °C for 48 h (protocol C), or 28 °C for 48 h (protocol D). Plates were incubated aerobically in protocols A, C, and D and anaerobically in protocol B. Antimicrobial activity was scored by measuring the diameter of the inhibition zone where no pathogen growth was observed. This overlay assay was initially performed in 12-well plates and all *Burkholderia* isolates showing an inhibition zone diameter of ≥5 mm in this format were considered active and were re-examined in 90 mm petri dishes to confirm the results.

### 4.3. Extraction and Identification of Specialized Metabolites

For the extraction and identification of specialized metabolites produced by selected *Burkholderia* strains with activity against Gram-negative pathogens, dense suspensions were prepared from fresh *Burkholderia* growth as described above. Four BSM-G agar plates were inoculated per *Burkholderia* strain with 100 µL of this suspension, left to dry, and incubated at 28 °C for 4 days. Cell material was removed from the agar surface of each BSM-G agar plate, and for each plate, the agar was ground and transferred to a 50 mL tube (Corning, USA). The ground agar was then extracted with an equal volume of methanol (20 mL) for 2 h at 220 rpm in an extraction hood cabinet. After centrifugation at 3000 rpm, the supernatant was transferred to a glass flask, and the four supernatants obtained per *Burkholderia* strain were pooled. The solvent was evaporated to dryness under a heated nitrogen stream and finally lyophilized to remove residual water. Five mg of the dried crude extract was resuspended in 200 µL of a 1:1 methanol:water mixture and filtered (0.45 µm pore size) into HPLC vials. The remaining material was stored at −20 °C.

Crude extracts were dereplicated by LC-HRMS as described previously [57] using an Agilent 1200 Rapid Resolution HPLC interfaced to a Bruker maXis mass spectrometer (Bruker Daltonics, Germany). A Waters Atlantis T3 column (4.6 × 100 mm, 5 µm particle size) was used for the separation. Two solvents were used as mobile phase: Solvent A (water:acetonitrile 90:10) and solvent B (water:acetonitrile 10:90), both with 13 mm ammonium formate and 0.01% trifluoroacetic acid. The mass spectrometer was operated in positive ESI mode. In one case, a signal was observed in the HPLC profile (UV trace at 210 nm), but no ionization was achieved in positive mode. The mass spectrometer was then operated in negative mode, but no ionization was observed either. The exact mass and the predicted molecular formula were used to search the DNP database to identify molecules. If a plausible match was found, considering the exact mass/molecular formula, the UV maxima, and the producing microorganism, the molecule was reported as a suggested component of the extract. If no match was found in the DNP, the predicted molecular formula was searched for in the PubChem [58] and ChemSpider [59] databases. Compounds for which no match was found in the above databases were considered potentially novel.

### 4.4. Semipreparative Fractionation of Crude Extracts and Dereplication of Active Fractions

Crude extracts of 14 *Burkholderia* isolates (Table 3) were prepared as described above. Extracts were dissolved in 100% dimethyl sulfoxide (DMSO) and filtered (0.45 µm pore size) prior to semipreparative fractionation using a Gilson GX-281 322H2 (Gilson Technologies, Hayward, CA, USA). Each extract was subjected to semipreparative reversed-phase HPLC (Zorbax SB-C18 column, 9.4 × 250 mm, 5 µm, 3.6 mL/min, UV detection at 210 and 280 nm) eluting with acetonitrile/water, in a linear gradient from 5 to 100% acetonitrile in 45 min, yielding 1.8 mL per fraction every 0.5 min to generate 80 central fractions. After evaporation of the organic solvent and redissolution in 20% DMSO in water, fractions were tested for antimicrobial activity against *A. baumannii* LMG 10520 and *C. freundii* R-67508 in duplicate on different days, as described earlier [60]. In brief, overnight cultures of both pathogens were grown in Tryptone Soya Broth (TSB; Oxoid, UK) and incubated at 37 °C with shaking at 220 rpm. The resulting cultures were then diluted to obtain assay inocula of approximately 1 × 10^5^ CFU/mL. For the assay, 90 μL/well of the diluted inoculum was mixed with 10 μL/well of each fraction. Polymyxin B and aztreonam (for *A. baumannii*) and polymyxin B and erythromycin (for *C. freundii*) were included as internal positive and negative controls for growth inhibition, respectively. All fractions resulting in ≥40% growth inhibition of at least one of the two tested pathogens and corresponding to a visible peak in the UV spectrum were subjected to LC-HRMS as described above to identify the specialized metabolites present in the active fractions.

## 5. Conclusions

*Burkholderia* bacteria have a large genomic potential for specialized metabolite biosynthesis and have been proposed as new and promising sources of novel antibiotics. The present study explored the potential for antibacterial activity towards important human pathogens of a taxonomically and geographically diverse collection of *Burkholderia* isolates. Initial dereplication of crude spent agar extracts of *Burkholderia* isolates that inhibited Gram-negative bacterial pathogens revealed the presence of 19 potentially novel metabolites. Semipreparative fractionation of the extracts with putative novel metabolites and activity testing of the resulting fractions showed that only one of those 19 potentially novel metabolites was present in a fraction that inhibited *A. baumannii* LMG 10520 and *C. freundii* R-67508; the remaining 18 putative novel metabolites were either not active against Gram-negative bacterial pathogens or were unstable. Although it is possible that the latter 18 putative novel metabolites were responsible for some of the antagonistic activity observed towards Gram-positive bacterial, and fungal pathogens, they might also have other interesting biological activities. The sensitivity of the initial dereplication of the crude extracts proved insufficient to detect four out of five putative novel metabolites that were detected in active fractions after fractionation. Semipreparative fractionation of interesting crude extracts, combined with activity testing of the fractions, provided a more detailed picture of the inhibitory potential of *Burkholderia* isolates. Further purification and structure elucidation will confirm the novelty of these compounds and determine whether they indeed represent new scaffolds. In addition, MIC and cytotoxicity tests will be essential to estimate the potential of these compounds for use as antimicrobial drugs.

Our results show that *Burkholderia* bacteria, and *Burkholderia* sensu stricto, in particular, are rich sources of potentially novel metabolites that are readily produced under standard laboratory conditions. The discovery of a total of 23 putative novel metabolites, five of which were present in fractions inhibiting the Gram-negative bacterial pathogens *A. baumannii* LMG 10520 and *C. freundii* R-67508, confirms and extends the observation that *Burkholderia* bacteria present exciting opportunities for the discovery of novel bioactive molecules.

## Figures and Tables

**Table 1:** Collection of *Burkholderia* isolates screened for antimicrobial activity. Isolates were screened for antimicrobial activity against *Staphylococcus aureus* ATCC 29213, *Acinetobacter baumannii* LMG 10520, and *Candida albicans* SC5314 using an overlay assay. Antimicrobial activity was defined as an inhibition zone diameter ≥5 mm. Isolates were assigned to Other Bcc groups by means of multilocus sequence typing analysis as described previously [31]. CLIN, clinical; ENV, environmental.

Species	Number of Isolates	Number with Antimicrobial Activity (CLIN/ENV)		
	(CLIN/ENV)	*S. aureus*	*A. baumannii*	*C. albicans*
*Burkholderia aenigmatica*	3 (1/2)	0	0	0
*Burkholderia ambifaria*	12 (3/8) ^1^	8 (2/6)	3 (0/3)	8 (3/5)
*Burkholderia anthina*	11 (2/9)	0	0	0
*Burkholderia arboris*	17 (10/7)	3 (0/3)	0	10 (3/7)
*Burkholderia catarinensis*	1 (0/1)	0	0	1 (0/1)
*Burkholderia cenocepacia*	12 (8/4)	2 (2/0)	1 (1/0)	5 (5/0)
*Burkholderia cepacia*	10 (5/5)	6 (3/3)	1 (0/1)	3 (1/2)
*Burkholderia contaminans*	16 (13/3)	1 (1/0)	0	9 (2/7)
*Burkholderia diffusa*	2 (1/1)	0	0	0
*Burkholderia dolosa*	1 (1/0)	0	0	0
*Burkholderia lata*	7 (2/5)	4 (2/2)	0	3 (1/2)
*Burkholderia latens*	1 (1/0)	0	0	0
*Burkholderia metallica*	1 (1/0)	0	0	0
*Burkholderia multivorans*	2 (2/0)	0	0	0
*Burkholderia pseudomultivorans*	1 (1/0)	0	0	0
*Burkholderia puraquae*	1 (0/1)	0	0	0
*Burkholderia pyrrocinia*	10 (2/8)	1 (0/1)	0	4 (0/4)
*Burkholderia seminalis*	1 (1/0)	0	0	0
*Burkholderia stabilis*	1 (1/0)	0	0	0
*Burkholderia stagnalis*	1 (0/1)	0	0	0
*Burkholderia territorii*	1 (0/1)	0	0	0
*Burkholderia ubonensis*	1 (0/1)	0	0	0
*Burkholderia vietnamiensis*	10 (2/8)	6 (1/5)	0	8 (1/7)
Other Bcc D	3 (3/0)	1 (1/0)	0	1 (1/0)
Other Bcc E	1 (0/1)	0	0	1 (0/1)
Other Bcc I	8 (1/7)	7 (1/6)	7 (1/6)	8 (1/7)
Other Bcc J	1 (1/0)	0	0	0
Other Bcc N	1 (0/1)	1 (0/1)	1 (0/1)	1 (0/1)
Other Bcc	1 (1/0)	0	0	1 (1/0)
*Burkholderia gladioli*	11 (5/5) ^1^	5 (2/2) ^1^	3 (1/2)	4 (2/2)
*Burkholderia glumae*	15 (1/14)	10 (0/10)	2 (0/2)	0
*Burkholderia plantarii*	8 (1/7)	8 (1/7)	0	2 (1/1)
*Burkholderia oklahomensis*	1 (1/0)	1 (1/0)	0	0
*Burkholderia singularis*	2 (2/0)	1 (1/0)	1 (1/0)	1 (1/0)
*Burkholderia thailandensis*	1 (0/1)	1 (0/1)	0	0
*Paraburkholderia bryophila*	6 (0/6)	1 (0/1)	0	1 (0/1)
*Paraburkholderia phenazinium*	1 (0/1)	1 (0/1)	0	1 (0/1)
*Paraburkholderia terricola*	6 (0/6)	1 (0/1)	0	0
*Paraburkholderia* spp.	39 (0/39)	0	0	0
*Caballeronia* spp.	25 (2/23)	0	0	0
*Robbsia andropogonis*	1 (0/1)	0	0	0
*Trinickia caryophyllii*	5 (0/5)	3 (0/3)	0	3 (0/3)
*Trinickia* spp.	2 (0/2)	0	0	0
*Mycetohabitans* spp.	2 (0/2)	0	0	0
Total isolates	263 (75/186) ^1^	72 (18/53)	19(4/15)	75 (23/52)

^1^ Isolation source is unknown.

**Table 2:** Spectrum of antimicrobial activity of 27 *Burkholderia* isolates. Antimicrobial activity was defined as an inhibition zone diameter ≥5 mm. +, activity; − no activity; Sa, *Staphylococcus aureus*; Ab, *Acinetobacter baumannii*; Ca, *Candida albicans*; Ef, *Enterococcus faecium*; Cd, *Clostridioides difficile*; Pa, *Pseudomonas aeruginosa*; Eb, *Enterobacter* spp.; Kp, *Klebsiella pneumoniae*; Cf, *Citrobacter freundii*; Ec, *Escherichia coli*; Mm, *Morganella morganii;* Cg, *Candida glabrata*; Ck, *Candida krusei*; Af, *Aspergillus fumigatus*. For all pathogens, three strains were tested, except for Ec (two strains), and Cf and Mm (one strain each). Isolates were assigned to Other Bcc groups by means of multilocus sequence typing analysis as described previously [31].

	Results of First Screen			Number of Strains with Antimicrobial Activity												
	Sa	Ab	Ca													
Strain	ATCC 29213	LMG 10520	SC 50314	Sa	Ef	Cd	Ab	Pa	Eb	Kp	Cf	Ec	Mm	Cg	Ck	Af
*Burkholderia ambifaria* LMG 19182^T^ t1 ^1^	+	+	+	1	3	3	3	0	2	0	1	2	1	3	3	3
*Burkholderia ambifaria* LMG 19182^T^ t2 ^1^	+	+	−	3	2	3	2	0	0	0	1	1	1	0	0	0
***Burkholderia ambifaria* R-50209**	+	+	+	2	3	3	2	0	2	0	1	2	1	3	3	3
***Burkholderia cenocepacia* R-1474**	+	+	+	3	3	3	3	0	3	3	1	2	1	2	1	2
*Burkholderia cenocepacia* R-49069	−	−	+	2	1	3	0	0	0	0	0	0	0	0	3	2
**Other Bcc I R-12632**	+	+	+	3	3	3	2	0	3	3	1	2	1	1	0	3
Other Bcc I R-14268	+	+	+	3	3	3	2	0	3	3	1	2	1	1	0	3
**Other Bcc I R-14280**	+	+	+	3	3	3	2	0	3	3	1	2	1	2	0	3
Other Bcc I R-14352	+	+	+	3	3	3	1	0	3	3	1	2	1	1	0	3
Other Bcc I R-14356	+	+	+	3	3	3	1	0	3	3	1	2	1	1	0	3
**Other Bcc I R-10741**	+	+	+	3	3	2	0	0	3	3	1	2	1	1	1	3
**Other Bcc I R-52250**	+	+	+	3	3	3	2	0	3	3	1	2	1	0	0	0
**Other Bcc N R-52245**	+	+	+	3	3	3	1	0	2	2	1	2	1	0	0	0
*Burkholderia cepacia* R-49076	+	−	+	3	1	0	1	0	0	0	0	0	0	2	3	3
***Burkholderia cepacia* R-24575**	+	−	−	1	3	0	0	0	0	1	1	2	1	0	0	2
***Burkholderia cepacia* LMG 1222^T^**	+	+	+	3	3	3	2	0	3	3	1	2	1	2	0	0
*Burkholderia arboris* R-8833	−	−	+	0	0	0	0	0	0	0	0	0	0	3	3	3
*Burkholderia vietnamiensis* R-24454	+	−	+	2	3	2	0	0	0	0	0	0	1	3	3	3
*Burkholderia gladioli* R-11809	+	−	+	3	3	3	0	0	0	0	1	0	1	3	3	3
*Burkholderia gladioli* LMG 2216^T^	+	+	+	3	3	3	2	0	1	0	0	0	0	1	0	3
***Burkholderia gladioli* R-16098**	+	+	+	3	3	3	3	0	0	0	0	2	0	0	0	0
***Burkholderia gladioli* R-20794**	−	+	−	2	3	3	2	0	0	0	0	0	1	0	1	0
***Burkholderia glumae* R-1678**	+	+	−	3	3	3	1	0	2	0	1	2	1	0	0	0
***Burkholderia glumae* R-8618**	+	−	−	2	1	2	0	0	2	0	1	2	1	0	0	0
*Burkholderia glumae* LMG 2196^T^	+	+	−	3	3	2	2	0	1	0	1	0	1	0	0	0
*Burkholderia plantarii* LMG 10908	+	−	−	3	2	3	0	0	0	0	0	0	0	0	0	0
***Burkholderia singularis* LMG 28155**	+	+	+	3	3	3	0	0	1	0	1	0	0	1	2	2

^1^ Strain LMG 19182 appeared as two distinct colony types. *Burkholderia* strains indicated in bold type were further analyzed through semipreparative fractionation.

**Table 3:** Specialized metabolites detected in crude extracts of 14 *Burkholderia* isolates. +, detected in the crude extract; −, not detected in the crude extract; cGMP, cyclic guanosine monophosphate. Isolates selected for semipreparative fractionation are indicated in bold type.

Molecular Formula	Common Name	R-50209	R-16098	R-20794	R-1678	R-8618	R-24575	LMG 1222^T^	R-12632	R-10741	R-14280	R-52250	R-52245	R-1474	LMG 28155
C_33_H_45_Cl_2_NO_11_	Enacyloxin	+	+	+	−	−	−	−	−	−	−	−	−	−	−
C_7_H_7_N_5_O_2_	Toxoflavin	−	+	−	+	+	−	−	−	−	−	−	−	−	−
C_6_H_5_N_5_O_2_	Reumycin	−	−	−	+	+	−	−	−	−	−	−	−	−	−
C_14_H_20_Cl_2_N_2_O_6_	Bactobolin A	−	−	−	+	−	−	−	−	−	−	−	−	−	−
C_52_H_85_N_11_O_22_	Cepacidin A1	+	−	−	−	−	−	−	−	−	−	−	−	−	−
C_19_H_25_NO	Antibiotic SF 2420B	+	−	−	−	−	+	−	−	−	−	−	−	−	−
C_17_H_21_NO		+	−	−	−	−	+	−	−	−	−	−	−	−	−
C_10_H_12_N_5_O_7_P	cGMP	−	−	−	+	−	−	−	−	−	−	−	−	−	−
C_8_H_11_NO_3_		−	−	−	+	−	−	−	−	−	−	−	−	−	−
C_10_H_6_Cl_2_N_2_O_2_	Pyrrolnitrin	−	−	−	−	−	+	−	+	+	+	+	−	−	−
C_10_H_8_Cl_2_N_2_	Aminopyrrolnitrin	−	−	−	−	−	+	−	−	+	−	+	−	−	−
C_21_H_24_FeN_3_O_9_	Antibiotic BN-227-F	−	−	−	−	−	+	−	−	−	−	−	−	−	−
C_7_H_9_NO_3_	Cepabactin	−	−	−	−	−	+	+	−	−	−	−	−	−	−
C_10_H_7_NO_3_S	Aeruginoic acid	−	−	−	−	−	+	+	+	−	+	+	−	−	−
C_10_H_9_NO_3_S	Dihydroaeruginoic acid	−	−	−	−	−	−	+	+	−	+	+	−	−	−
C_10_H_11_NO_2_S	Aerugine	−	−	−	−	−	−	+	+	−	+	+	−	−	−
C_14_H_16_N_2_O_3_S_2_	Pyochelin	−	−	−	−	−	−	+	+	−	+	+	−	−	−
C_14_H_10_O_4_S	Ditropolonyl sulfide	−	−	−	−	−	−	+	−	+	+	+	+	+	−
C_30_H_56_N_8_O_13_	Ornibactin C8	−	−	−	−	−	−	+	+	+	+	+	+	+	−
C_28_H_52_N_8_O_13_	Ornibactin C6	−	−	−	−	−	−	−	+	+	+	+	+	+	−
C_26_H_48_N_8_O_13_	Ornibactin C4	−	−	−	−	−	−	−	+	−	−	−	+	+	−
C_11_H_8_Cl_2_N_2_O_4_		−	−	−	−	−	−	−	+	+	−	−	−	−	−
C_12_H_12_O_4_	Differolide	−	−	−	−	−	−	−	−	−	−	−	−	−	+
C_33_H_47_Cl_2_NO_13_^1^		+	−	+	−	−	−	−	−	−	−	−	−	−	−
C_36_H_64_N_4_O_10_^1^		−	+	−	−	−	−	−	−	−	−	−	−	−	−
C_17_H_27_Cl_2_NO_7_^1^		−	−	+	−	−	−	−	−	−	−	−	−	−	−
C_7_H_9_N_5_O_3_^1^		−	−	−	+	−	−	−	−	−	−	−	−	−	−
C_9_H_21_NO_3_^1^		−	−	−	+	−	−	−	−	−	−	−	−	−	−
C_17_H_25_N_3_O_13_^1^		−	−	−	+	−	−	−	−	−	−	−	−	−	−
C_10_H_9_NO_2_^1^		−	−	−	−	−	+	−	−	−	−	−	−	−	−
C_19_H_13_N_5_O_12_S ^1^		−	−	−	−	−	+	−	−	−	−	−	−	−	−
C_9_H_9_NO_3_^1^		−	−	−	−	−	−	−	+	−	−	−	−	−	−
C_15_H_12_O_6_S ^1^		−	−	−	−	−	−	−	+	−	−	−	−	−	−
C_18_H_33_N_3_O_12_^1^		−	−	−	−	−	−	−	+	−	−	−	−	−	−
C_26_H_42_O_7_^1^		−	−	−	−	−	−	−	+	−	−	−	−	−	−
C_35_H_39_N_9_O_11_^1^		−	−	−	−	−	−	−	+	−	−	−	+	+	−
C_37_H_43_N_9_O_11_^1^		−	−	−	−	−	−	−	+	−	−	−	+	+	−
C_26_H_48_N_8_O_14_^1^		−	−	−	−	−	−	−	−	−	−	−	+	+	−
C_33_H_35_N_9_O_11_^1^		−	−	−	−	−	−	−	−	−	−	−	+	+	−
C_21_H_25_FeNO_15_S_2_^1^		−	−	−	−	−	−	−	−	−	+	−	−	−	−
C_14_H_14_O_3_^1^		−	−	−	−	−	−	−	−	−	−	−	−	−	+
C_16_H_22_O_3_^1^		−	−	−	−	−	−	−	−	−	−	−	−	−	+

^1^ Putative novel molecules not present in the Chapman and Hall Dictionary of Natural Products.

**Table 4:** Specialized metabolites detected in active fractions of six *Burkholderia* isolates. Crude agar extracts were subjected to semipreparative fractionation and tested for antimicrobial activity against *Citrobacter freundii* R-67508 and *Acinetobacter baumannii* LMG 10520. Metabolites present within active fractions resulting in ≥40% growth reduction of at least one of the two pathogens were identified using LC-HRMS.

Strain	Fraction	Metabolite	Growth Reduction (%)	
			*C. freundii*	*A. baumannii*
*Burkholderia ambifaria* R-50209				
	36	Enacyloxin IIa or IIb	100	100
	38	Enacyloxin IIa or IIb	100	99
	51	Pyrrolnitrin	7	38
	57	C_17_H_21_NO	5	70
	67	Antibiotic SF 2420B	10	44
*Burkholderia gladioli* R-16098				
	15	Reumycin	47	55
	40	C_33_H_47_Cl_2_NO_13_^1^	98	100
	42	Enacyloxin IIa or IIb	89	100
	43	Enacyloxin IIa or IIb	78	99
*Burkholderia glumae* R-1678				
	14	Reumycin	100	96
	15	Reumycin	100	95
	20	Toxoflavin	64	86
	22	Bactobolin A	100	100
	23	C_16_H_23_Cl_2_N_3_O_6_^1^	100	99
	26	C_16_H_22_Cl_2_N_2_O_7_^1^	93	55
	27	C_16_H_22_Cl_2_N_2_O_7_^1^	100	90
*Burkholderia cepacia* R-24575				
	22	Cepabactin	100	99
		Antibiotic BN-227-F		
	51	Pyrrolnitrin	18	43
	58	C_17_H_21_NO	10	55
	68	Antibiotic SF 2420B	11	61
Other Bcc I R-12632				
	31	Ditropolonyl sulfide	99	97
		C_47_H_61_N_3_O_16_^1^		
	51	Pyrrolnitrin	39	36
Other Bcc I R-14280				
	28	Ditropolonyl sulfide	98	97
	38	Ornibactin C6	73	99
		C_10_H_11_NO_2_S_3_		
		C_9_H_9_NO_2_S ^1^		
	39	Ornibactin C6	70	88
		Aerugine		
		Aeruginol		
	51	Ornibactin C8	54	72
		Pyrrolnitrin		
	54	Ornibactin C8	52	22

^1^ Putative novel molecules not present in the Chapman and Hall Dictionary of Natural Products.

## Data Availability

The data presented in this study are available in Supplementary Figures S1–S3.

## Supplementary Materials

The following are available online at https://www.mdpi.com/2079-6382/10/2/147/s1, Figure S1: Mass spectra and UV spectra of 42 metabolites detected in crude extracts of 14 *Burkholderia* isolates title, Figure S2: Antimicrobial activity of semipreparative fractions of *Burkholderia gladioli* R-20794 and *Burkholderia singularis* LMG 28155, Figure S3: Semipreparative fractionation chromatograms of eight *Burkholderia* isolates. Table S1: *Burkholderia* strains included in the present study title, Table S2: Pathogens used in antimicrobial activity assays.

## References

[1] IACG (2019). No Time to Wait: Securing the Future from Drug-Resistant Infections—Report to the Secretary-General of the United Nations. https://www.who.int/antimicrobial-resistance/interagency-coordination-group/final-report/en/.

[2] Pendleton J.N., Gorman S.P., Gilmore B.F. (2013). Clinical relevance of the ESKAPE pathogens. Expert Rev. Anti. Infect. Ther..

[3] Spížek J., Novotná J., Rezanka T., Demain A.L. (2010). Do we need new antibiotics? The search for new targets and new compounds. J. Ind. Microbiol. Biotechnol..

[4] Demain A.L. (2014). Importance of microbial natural products and the need to revitalize their discovery. J. Ind. Microbiol. Biotechnol..

[5] Genilloud O. (2017). Actinomycetes: Still a source of novel antibiotics. Nat. Prod. Rep..

[6] Depoorter E., Bull M.J., Peeters C., Coenye T., Vandamme P., Mahenthiralingam E. (2016). Burkholderia: An update on taxonomy and biotechnological potential as antibiotic producers. Appl. Microbiol. Biotechnol..

[7] Kunakom S., Eustáquio A.S. (2019). Burkholderia as a Source of Natural Products. J. Nat. Prod..

[8] Esmaeel Q., Pupin M., Jacques P., Leclère V. (2018). Nonribosomal peptides and polyketides of Burkholderia: New compounds potentially implicated in biocontrol and pharmaceuticals. Environ. Sci. Pollut. Res..

[9] Mahenthiralingam E., Urban T.A., Goldberg J.B. (2005). The multifarious, multireplicon Burkholderia cepacia complex. Nat. Rev. Microbiol..

[10] Eberl L., Vandamme P. (2016). Members of the genus Burkholderia: Good and bad guys. F1000Research.

[11] Sawana A., Adeolu M., Gupta R.S. (2014). Molecular signatures and phylogenomic analysis of the genus Burkholderia: Proposal for division of this genus into the emended genus Burkholderia containing pathogenic organisms and a new genus Paraburkholderia gen. nov. harboring env. Front. Genet..

[12] Dobritsa A.P., Samadpour M. (2016). Transfer of eleven species of the genus Burkholderia to the genus Paraburkholderia and proposal of Caballeronia gen. nov. to accommodate twelve species of the genera Burkholderia and Paraburkholderia. Int. J. Syst. Evol. Microbiol..

[13] Lopes-Santos L., Castro D.B.A., Ferreira-Tonin M., Corrêa D.B.A., Weir B.S., Park D., Ottoboni L.M.M., Neto J.R., Destéfano S.A.L. (2017). Reassessment of the taxonomic position of Burkholderia andropogonis and description of Robbsia andropogonis gen. nov., comb. nov. Antonie Leeuwenhoek.

[14] Estrada-de los Santos P., Palmer M., Chávez-Ramírez B., Beukes C., Steenkamp E.T., Briscoe L., Khan N., Maluk M., Lafos M., Humm E. (2018). Whole genome analyses suggests that Burkholderia sensu lato contains two additional novel genera (Mycetohabitans gen. nov., and Trinickia gen. nov.): Implications for the evolution of diazotrophy and nodulation in the Burkholderiaceae. Genes.

[15] Marolda C.L., Hauröder B., John M.A., Michel R., Valvano M.A. (1999). Intracellular survival and saprophytic growth of isolates from the Burkholderia cepacia complex in free-living amoebae. Microbiology.

[16] Partida-Martinez L.P., Groth I., Schmitt I., Richter W., Roth M., Hertweck C. (2007). Burkholderia rhizoxinica sp. nov. and Burkholderia endofungorum sp. nov., bacterial endosymbionts of the plant-pathogenic fungus Rhizopus microsporus. Int. J. Syst. Evol. Microbiol..

[17] Currie B.J. (2010). Burkholderia pseudomallei and Burkholderia mallei. Mandell, Douglas, and Bennett’s Principles and Practice of Infectious Diseases.

[18] Vial L., Chapalain A., Groleau M.C., Déziel E. (2011). The various lifestyles of the *Burkholderia cepacia* complex species: A tribute to adaptation. Environ. Microbiol..

[19] Suárez-Moreno Z.R., Caballero-Mellado J., Coutinho B.G., Mendonça-Previato L., James E.K., Venturi V. (2012). Common features of environmental and potentially beneficial plant-associated Burkholderia. Microb. Ecol..

[20] Mahenthiralingam E., Baldwin A., Dowson C.G. (2008). Burkholderia cepacia complex bacteria: Opportunistic pathogens with important natural biology. J. Appl. Microbiol..

[21] LiPuma J.J. (2010). The changing microbial epidemiology in cystic fibrosis. Clin. Microbiol. Rev..

[22] Azegami K., Nishiyama K., Watanabe Y., Kadota I., Ohuchi A., Fukazawa C. (1987). Pseudomonas plantarii sp. nov., the causal agent of rice seedling blight. Int. J. Syst. Bacteriol..

[23] Jeong Y., Kim J., Kim S., Kang Y., Nagamatsu T., Hwang I. (2003). Toxoflavin produced by Burkholderia glumae causing rice grain rot is responsible for inducing bacterial wilt in many field crops. Plant Dis..

[24] Parke J.L., Gurian-Sherman D. (2001). Diversity of the Burkholderia cepacia complex and implications for risk assessment of biological control strains. Annu. Rev. Phytopathol..

[25] Hwang J., Chilton W.S., Benson D.M. (2002). Pyrrolnitrin production by Burkholderia cepacia and biocontrol of Rhizoctonia stem rot of poinsettia. Biol. Control.

[26] Wang X.Q., Liu A.X., Guerrero A., Liu J., Yu X.Q., Deng P., Ma L., Baird S.M., Smith L., Li X.D. (2016). Occidiofungin is an important component responsible for the antifungal activity of Burkholderia pyrrocinia strain Lyc2. J. Appl. Microbiol..

[27] Mullins A.J., Murray J.A.H., Bull M.J., Jenner M., Jones C., Webster G., Green A.E., Neill D.R., Connor T.R., Parkhill J. (2019). Genome mining identifies cepacin as a plant-protective metabolite of the biopesticidal bacterium Burkholderia ambifaria. Nat. Microbiol..

[28] Mahenthiralingam E., Song L., Sass A., White J., Wilmot C., Marchbank A., Boaisha O., Paine J., Knight D., Challis G.L. (2011). Enacyloxins are products of an unusual hybrid modular polyketide synthase encoded by a cryptic Burkholderia ambifaria genomic island. Chem. Biol..

[29] Song L., Jenner M., Masschelein J., Jones C., Bull M.J., Harris S.R., Hartkoorn R.C., Vocat A., Romero-Canelon I., Coupland P. (2017). Discovery and Biosynthesis of Gladiolin: A Burkholderia gladioli Antibiotic with Promising Activity against Mycobacterium tuberculosis. J. Am. Chem. Soc..

[30] Wu Y., Seyedsayamdost M.R. (2018). The Polyene Natural Product Thailandamide A Inhibits Fatty Acid Biosynthesis in Gram-Positive and Gram-Negative Bacteria. Biochemistry.

[31] Spilker T., Baldwin A., Bumford A., Dowson C.G., Mahenthiralingam E., LiPuma J.J. (2009). Expanded Multilocus Sequence Typing for Burkholderia Species. J. Clin. Microbiol..

[32] Vandamme P., Peeters C. (2014). Time to revisit polyphasic taxonomy. Antonie Leeuwenhoek.

[33] Byng G.S., Turner J.M. (1975). Phenazine Biosynthesis by a Pseudomonad. Biochem. Soc. Trans..

[34] Vandamme P., Opelt K., Knochel N., Berg C., Schonmann S., De Brandt E., Eberl L., Falsen E., Berg G. (2007). Burkholderia bryophila sp. nov. and Burkholderia megapolitana sp. nov., moss-associated species with antifungal and plant-growth-promoting properties. Int. J. Syst. Evol. Microbiol..

[35] Gasser I., Cardinale M., Müller H., Heller S., Eberl L., Lindenkamp N., Kaddor C., Steinbüchel A., Berg G. (2011). Analysis of the endophytic lifestyle and plant growth promotion of Burkholderia terricola ZR2-12. Plant Soil.

[36] Baldwin A., Mahenthiralingam E., Kathleen M., Honeybourne D., Maiden M.C.J., John R., Speert D.P., Lipuma J.J., Vandamme P., Dowson C.G. (2005). Multilocus sequence typing scheme that provides both species and strain differentiation for the Burkholderia cepacia complex. J. Clin. Microbiol..

[37] Groenhagen U., Baumgartner R., Bailly A., Gardiner A., Eberl L., Schulz S., Weisskopf L. (2013). Production of bioactive volatiles by different *Burkholderia ambifaria* strains. J. Chem. Ecol..

[38] Meyer J.M., Hohnadel D., Hallé F. (1989). Cepabactin from Pseudomonas cepacia, a new type of siderophore. J. Gen. Microbiol..

[39] Itoh J., Miyadoh S., Takahasi S., Amano S., Ezaki N., Yamada Y. (1979). Studies on antibiotics BN-227 and BN-227-F., new antibiotics. I. Taxonomy, isolation and characterization. J. Antibiot. (Tokyo).

[40] Deng P., Foxfire A., Xu J., Baird S.M., Jia J., Delgado K.H., Shin R., Smith L., Lu S.-E. (2017). The Siderophore Product Ornibactin Is Required for the Bactericidal Activity of Burkholderia contaminans MS14. Appl. Environ. Microbiol..

[41] Adler C., Corbalán N.S., Seyedsayamdost M.R., Pomares M.F., de Cristóbal R.E., Clardy J., Kolter R., Vincent P.A. (2012). Catecholate Siderophores Protect Bacteria from Pyochelin Toxicity. PLoS ONE.

[42] Brüsewitz G., Molls W., Westphal C., Pulverer G. (1981). Substituted Tropolones, Process for the Preparation Thereof and Pharmaceutical Compositions Containing These.

[43] Lee J.Y., Moon S.S., Hwang B.K. (2003). Isolation and Antifungal and Antioomycete Activities of Aerugine Produced by Pseudomonas fluorescens Strain MM-B16. Appl. Environ. Microbiol..

[44] Carmi R., Carmeli S., Levy E., Gough F.J. (1994). (+)-(S)-Dihydroaeruginoic Acid, an Inhibitor of Septoria tritici and Other Phytopathogenic Fungi and Bacteria, Produced by Pseudomonas fluorescens. J. Nat. Prod..

[45] Ye L., Cornelis P., Guillemyn K., Ballet S., Christophersen C., Hammerich O. (2014). Structure revision of N-mercapto-4-formylcarbostyril produced by Pseudomonas fluorescens G308 to 2-(2-hydroxyphenyl)thiazole-4-carbaldehyde [aeruginaldehyde]. Nat. Prod. Commun..

[46] Trottmann F., Franke J., Ishida K., García-Altares M., Hertweck C. (2019). A Pair of Bacterial Siderophores Releases and Traps an Intercellular Signal Molecule: An Unusual Case of Natural Nitrone Bioconjugation. Angew. Chem. Int. Ed..

[47] Ross C., Opel V., Scherlach K., Hertweck C. (2014). Biosynthesis of antifungal and antibacterial polyketides by Burkholderia gladioli in coculture with Rhizopus microsporus. Mycoses.

[48] Kondo S., Horiuchi Y., Hamada M., Takeuchi T., Umezawa H. (1979). A new antitumor antibiotic, bactobolin produced by Pseudomonas. J. Antibiot. (Tokyo).

[49] Seyedsayamdost M.R., Chandler J.R., Blodgett J.A.V., Lima P.S., Duerkop B.A., Oinuma K.-I., Greenberg E.P., Clardy J. (2010). Quorum-sensing-regulated bactobolin production by Burkholderia thailandensis E264. Org. Lett..

[50] Li X., Li Y., Wang R., Wang Q., Lu L. (2019). Toxoflavin Produced by Burkholderia gladioli from Lycoris aurea Is a New Broad-Spectrum Fungicide. Appl. Environ. Microbiol..

[51] Ma J., Yoneda F., Nagamatsu T. (2015). Synthesis of 6-Azapurines by Transformation of Toxoflavins and Reumycins (7-Azapteridines) and their Cytotoxicities. Aust. J. Chem..

[52] Butt A.T., Thomas M.S. (2017). Iron Acquisition Mechanisms and Their Role in the Virulence of Burkholderia Species. Front. Cell. Infect. Microbiol..

[53] Schmidt S., Blom J.F., Pernthaler J., Berg G., Baldwin A., Mahenthiralingam E., Eberl L. (2009). Production of the antifungal compound pyrrolnitrin is quorum sensing-regulated in members of the Burkholderia cepacia complex. Environ. Microbiol..

[54] Serino L., Reimmann C., Visca P., Beyeler M., Della Chiesa V., Haas D. (1997). Biosynthesis of pyochelin and dihydroaeruginoic acid requires the iron-regulated pchDCBA operon in Pseudomonas aeruginosa. J. Bacteriol..

[55] Biggins J.B., Liu X., Feng Z., Brady S.F. (2011). Metabolites from the induced expression of cryptic single operons found in the genome of *Burkholderia pseudomallei*. J. Am. Chem. Soc..

[56] O’Sullivan L.A., Weightman A.J., Jones T.H., Marchbank A.M., Tiedje J.M., Mahenthiralingam E. (2007). Identifying the genetic basis of ecologically and biotechnologically useful functions of the bacterium Burkholderia vietnamiensis. Environ. Microbiol..

[57] Santos J.D., Vitorino I., De La Cruz M., Díaz C., Cautain B., Annang F., Pérez-Moreno G., Martinez I.G., Tormo J.R., Martín J.M. (2019). Bioactivities and extract dereplication of actinomycetales isolated from marine sponges. Front. Microbiol..

[58] Kim S., Thiessen P.A., Bolton E.E., Chen J., Fu G., Gindulyte A., Han L., He J., He S., Shoemaker B.A. (2016). PubChem substance and compound databases. Nucleic Acids Res..

[59] Pence H.E., Williams A. (2010). Chemspider: An online chemical information resource. J. Chem. Educ..

[60] Audoin C., Bonhomme D., Ivanisevic J., Cruz M., Cautain B., Monteiro M., Reyes F., Rios L., Perez T., Thomas O. (2013). Balibalosides, an Original Family of Glucosylated Sesterterpenes Produced by the Mediterranean Sponge Oscarella balibaloi. Mar. Drugs.

