# Supplementary material for: Burkholderia Bacteria Produce Multiple Potentially Novel Molecules that Inhibit Carbapenem-Resistant Gram-Negative Bacterial Pathogens
Source: Antibiotics (Basel). 2021 Feb 2;10(2):147. doi: 10.3390/antibiotics10020147 (PMC7912996; doi:10.3390/antibiotics10020147)

S1 Fig. Mass spectra and UV spectra of 42 metabolites detected in crude extracts of 14 *Burkholderia* isolates. The upper panel represents the UV spectrum, the lower panel represents the mass spectrum in positive mode. Asterisks indicate putative novel molecules.

$C_{33}H_{45}Cl_2NO_{11}$ – Enacyloxin IIa or IIb

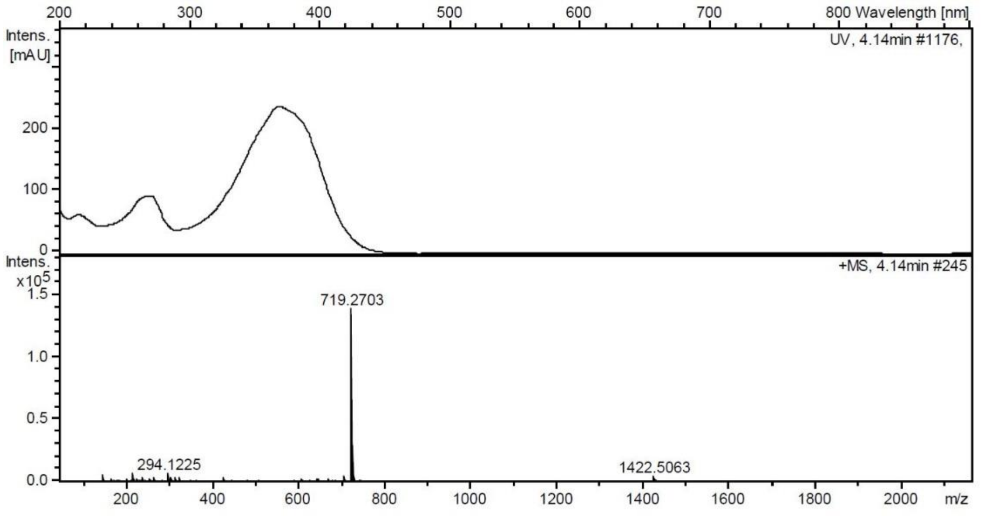

$C_7H_7N_5O_2$ – Toxoflavin

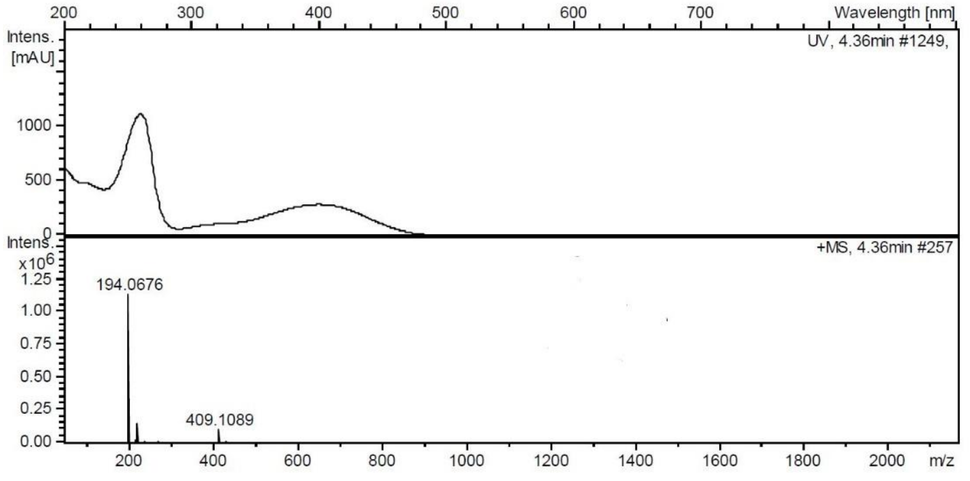

$C_6H_5N_5O_2$ – Reumycin

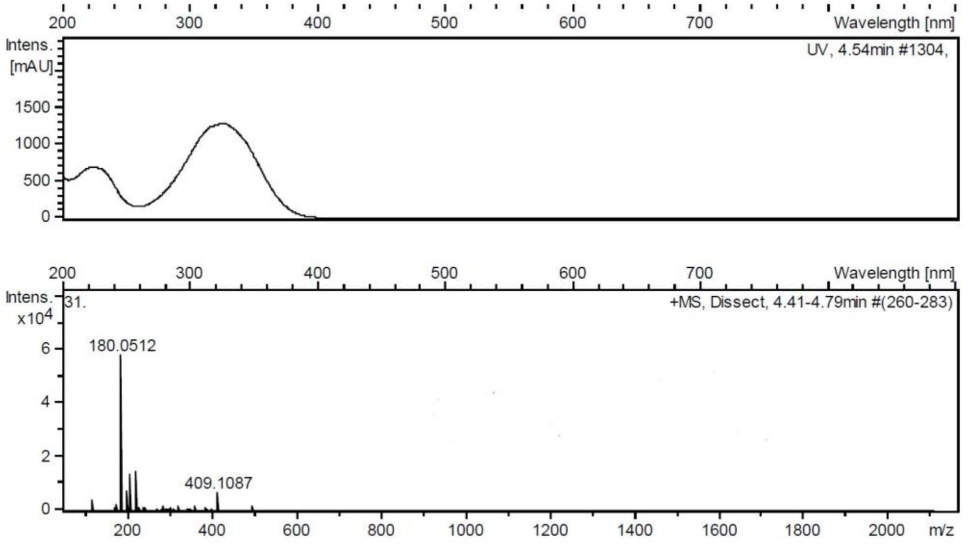

$C_{14}H_{20}Cl_2N_2O_6$ – Bactobolin A

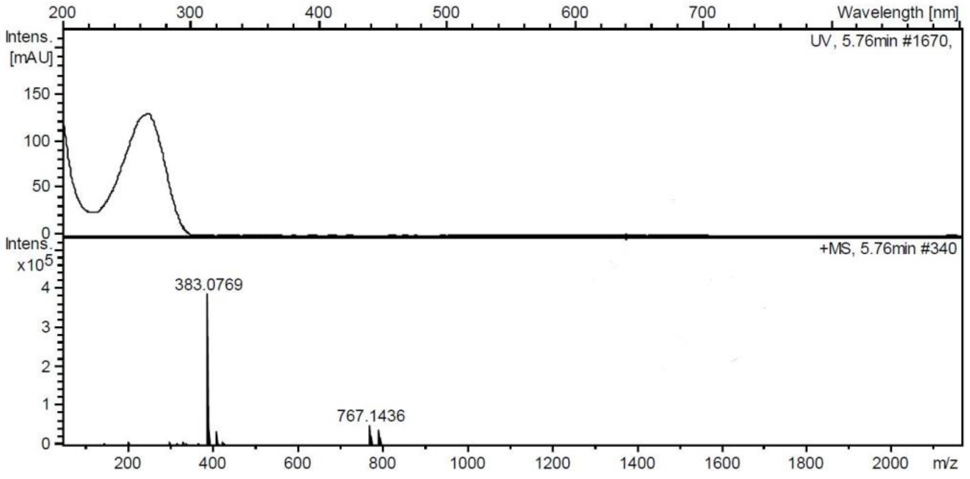

$C_{52}H_{85}N_{11}O_{22}$ – Cepacidin A1

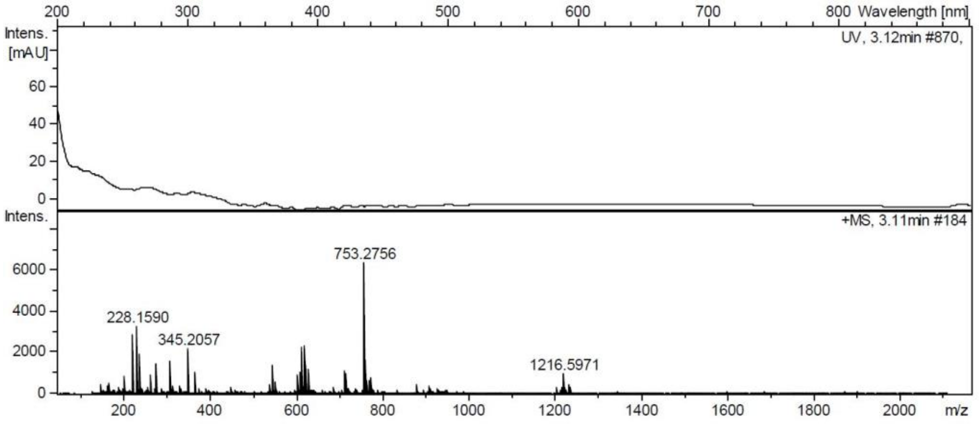

$C_{19}H_{25}NO$ – Antibiotic SF 2420B

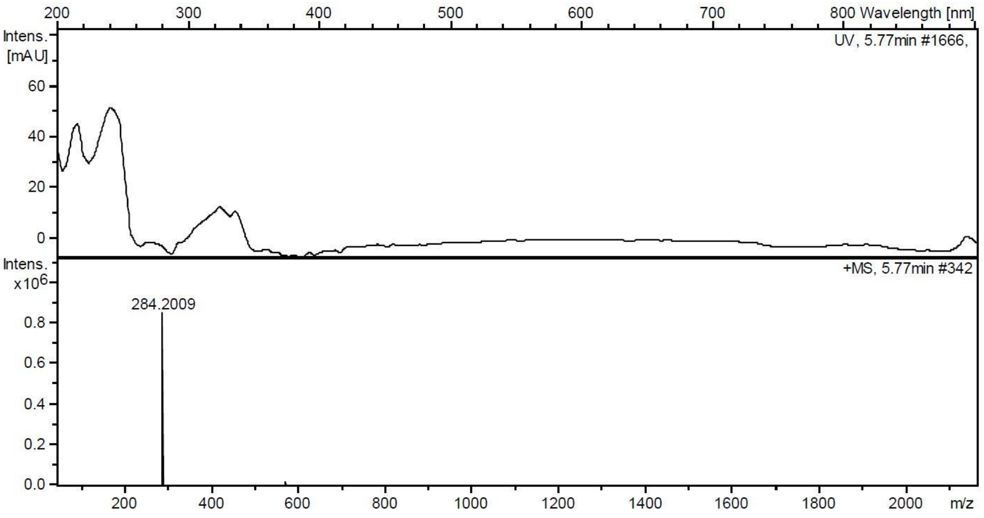

$C_{17}H_{21}NO$ – 2-(2-Heptenyl)-3-methyl-4(1H)-quinolinone

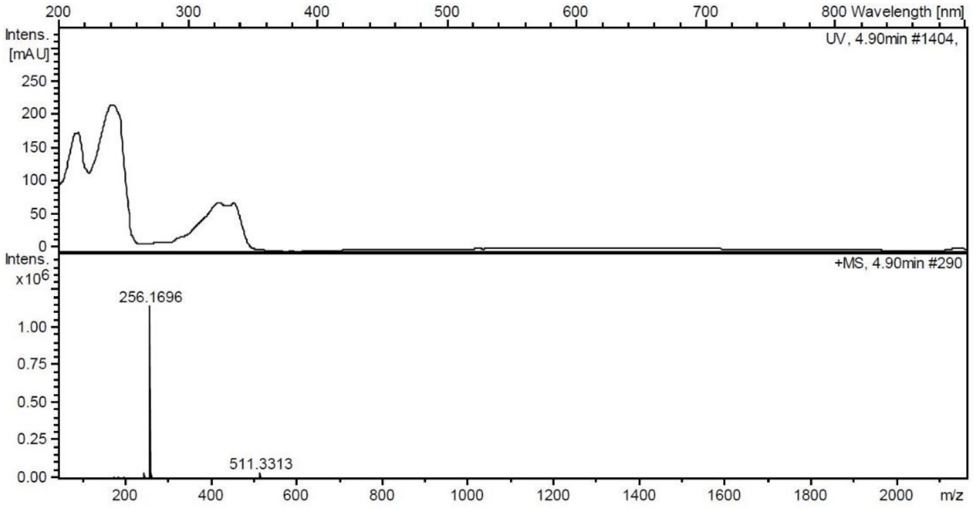

$C_{10}H_{12}N_5O_7P$ – Cyclic guanosine monophosphate (cGMP)

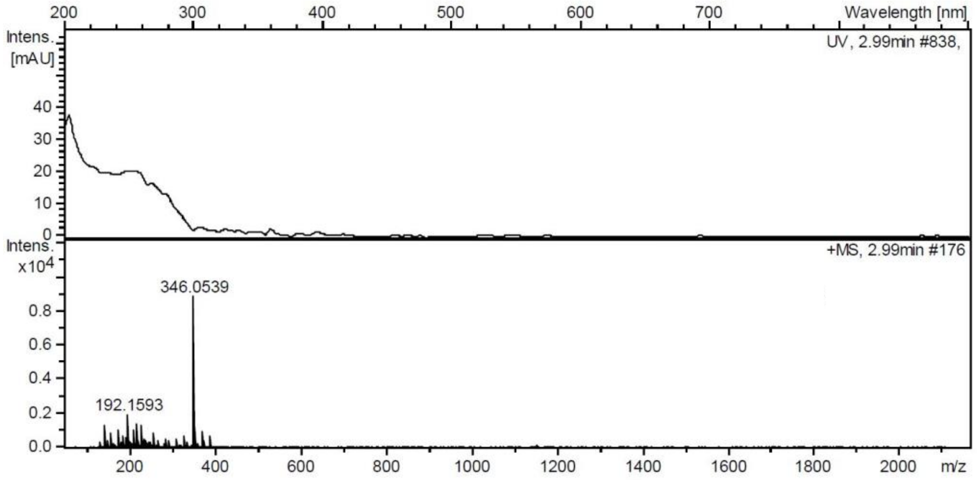

$C_8H_{11}NO_3$ – 2-Acetamido-4-hydroxy-2-cyclohexen-1-one

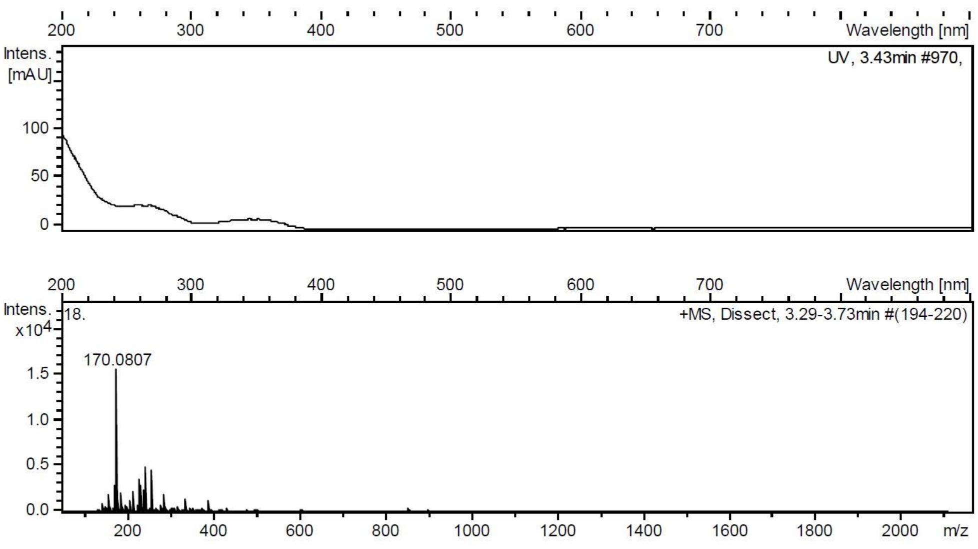

$C_{10}H_6Cl_2N_2O_2$ – Pyrrolnitrin

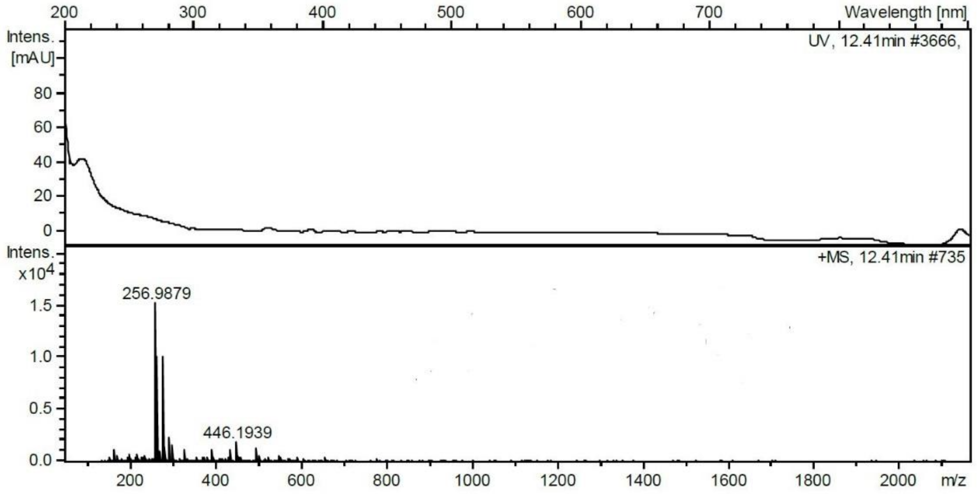

$C_{10}H_8Cl_2N_2$ – Aminopyrrolnitrin

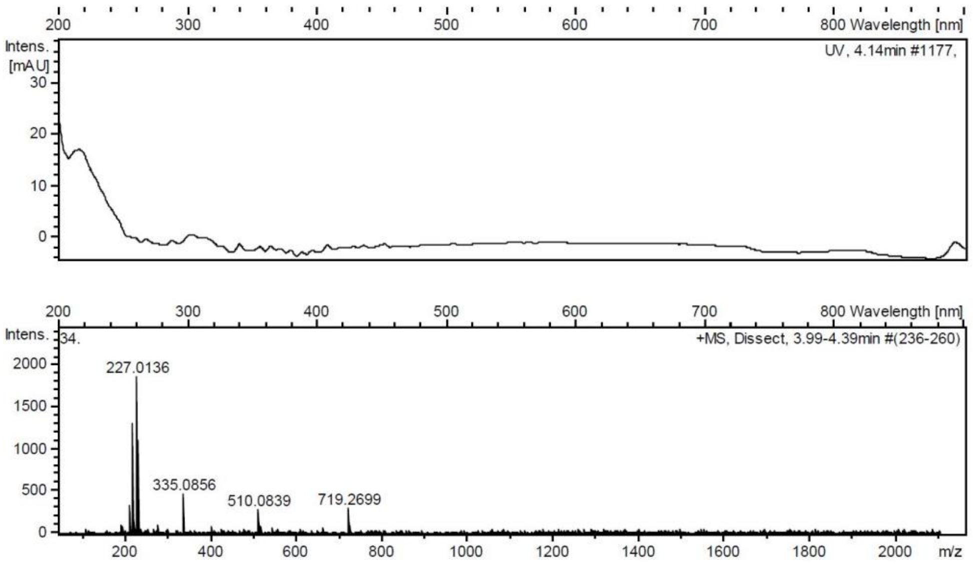

$C_{21}H_{24}FeN_3O_9$ – Antibiotic BN 227F

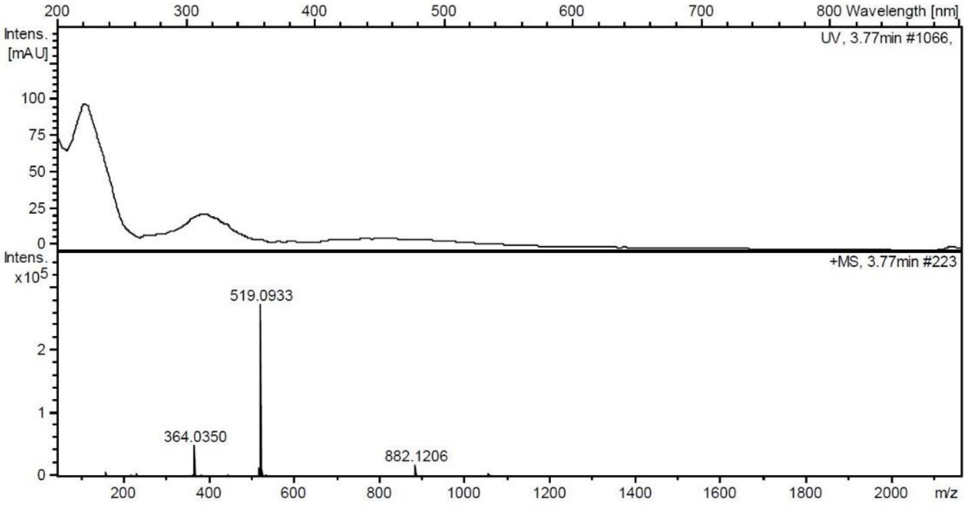

$C_7H_9NO_3$ – Cepabactin (= Antibiotic BN 227)

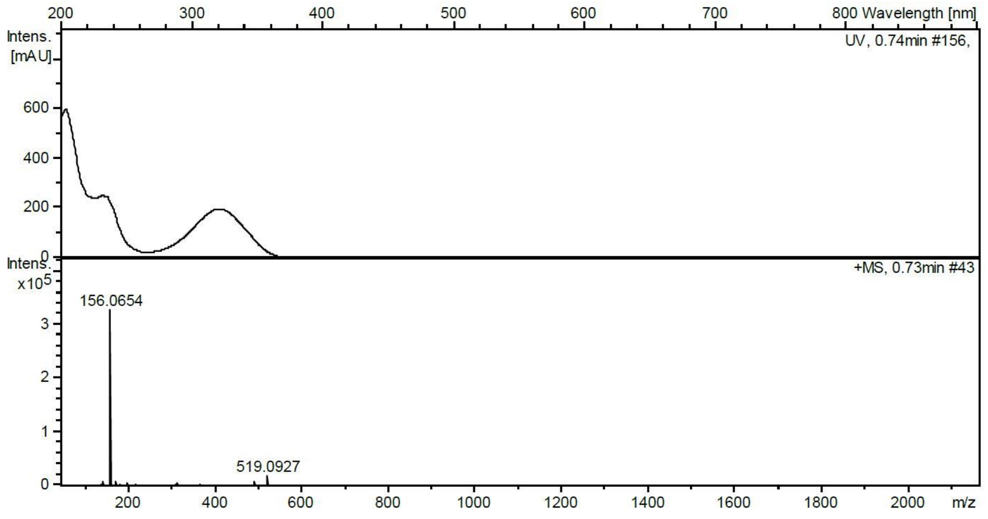

$C_{10}H_7NO_3S$ – Aeruginoic acid

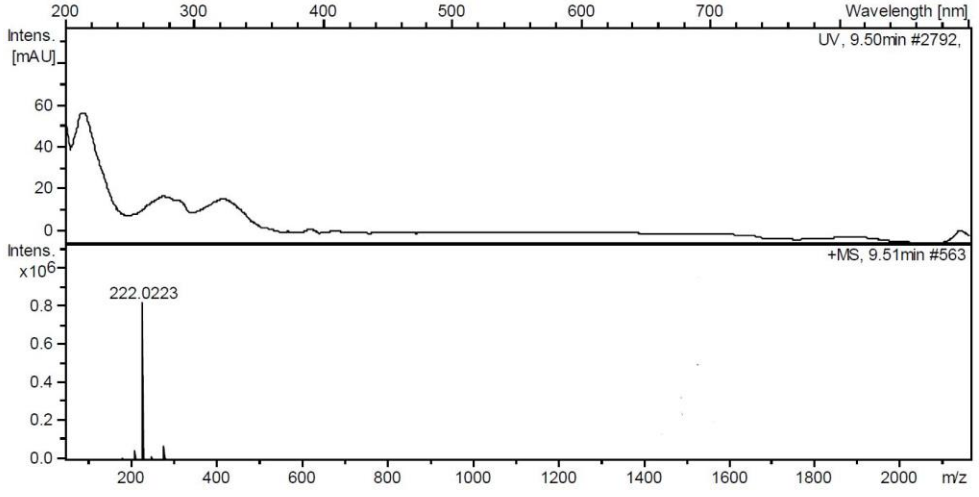

$C_{10}H_9NO_3S$ – Dihydroaeruginoic acid

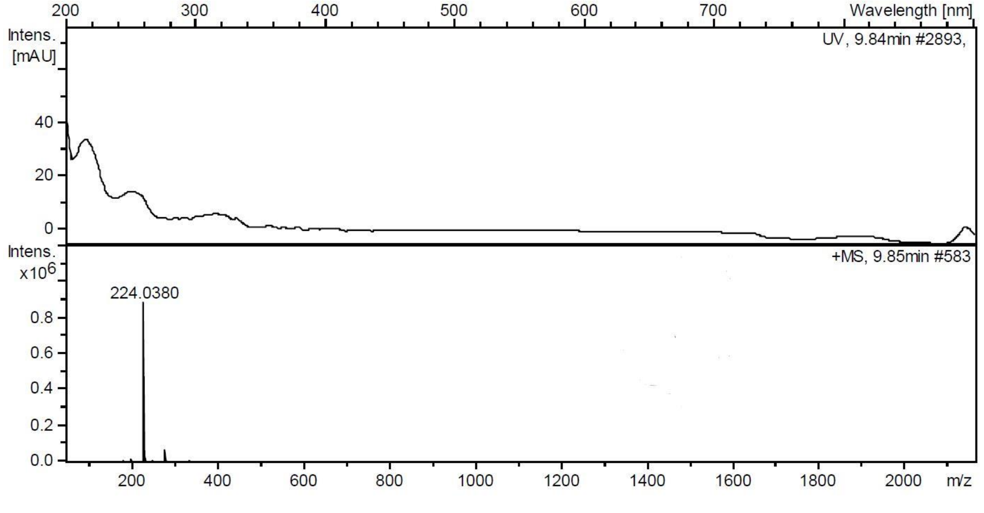

$C_{10}H_{11}NO_2S$ – Aerugine

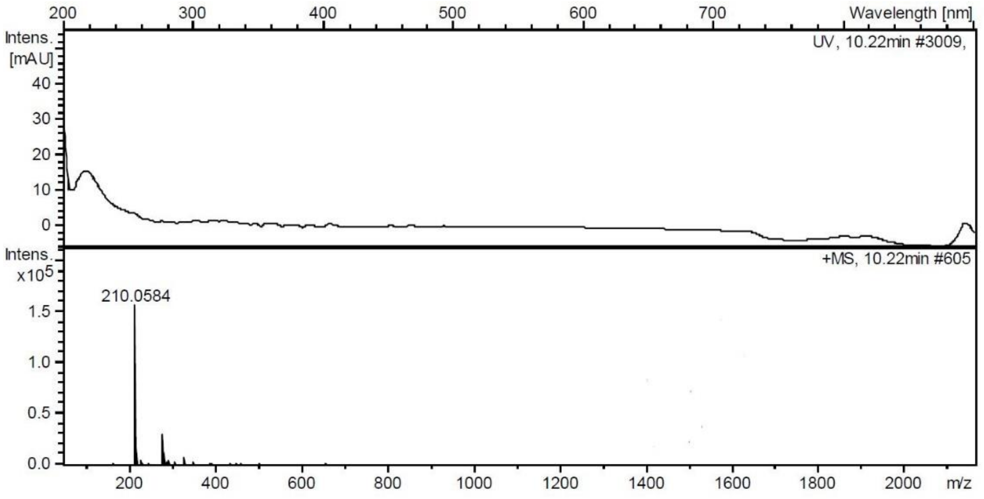

$C_{14}H_{16}N_2O_3S_2$ – Pyochelin

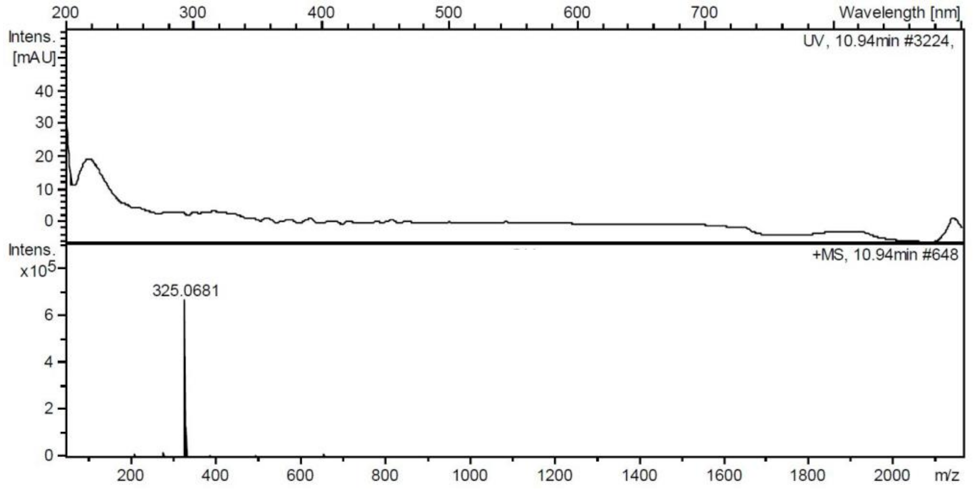

$C_{14}H_{10}O_4S$ – Ditropolonyl sulfide

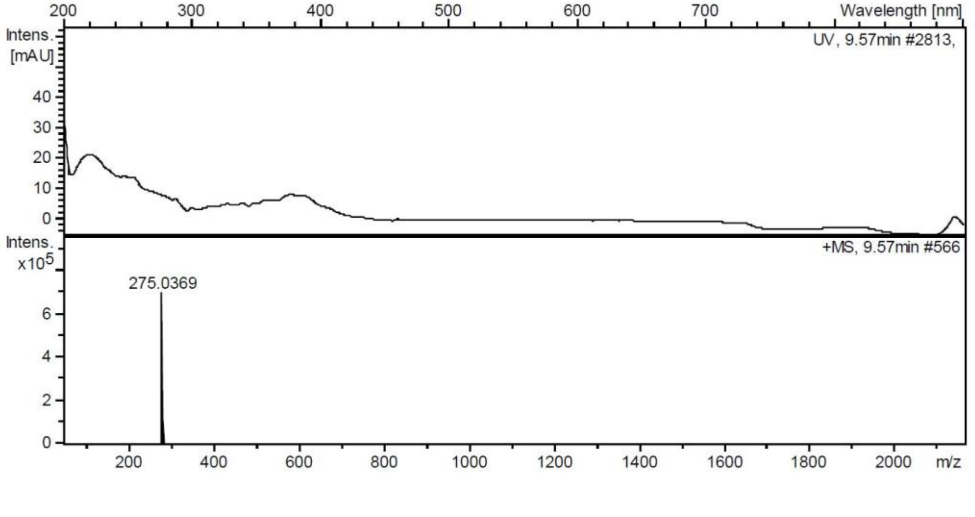

$C_{30}H_{56}N_8O_{13}$ – Ornibactin C8

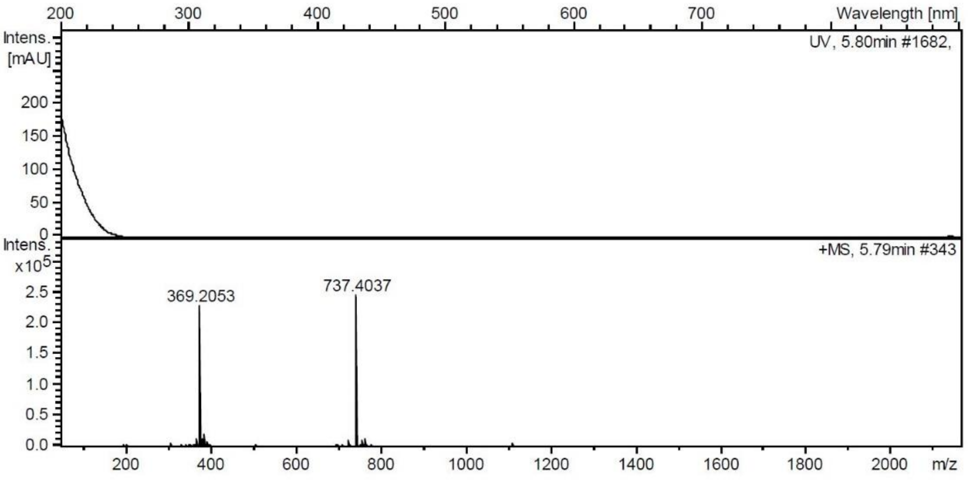

$C_{28}H_{52}N_8O_{13}$ – Ornibactin C6

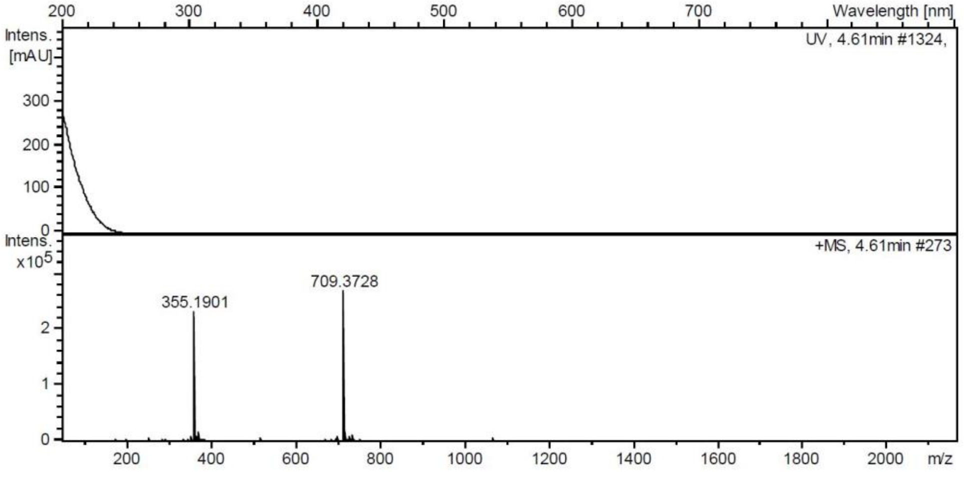

$C_{26}H_{48}N_8O_{13}$ – Ornibactin C4

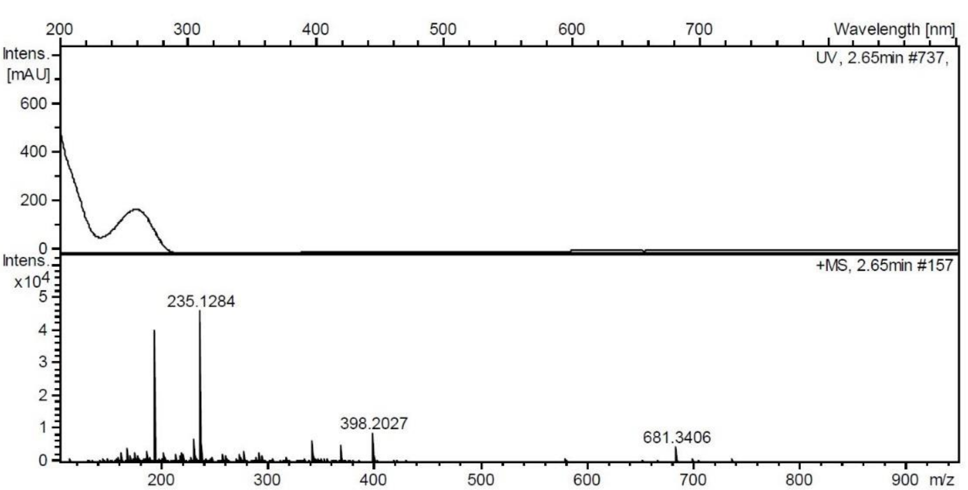

$C_{11}H_8Cl_2N_2O_4$ – 3-Chloro-4-(3-chloro-2-nitrophenyl)-1,5-dihydro-5-methoxy-2H-pyrrol-2-one

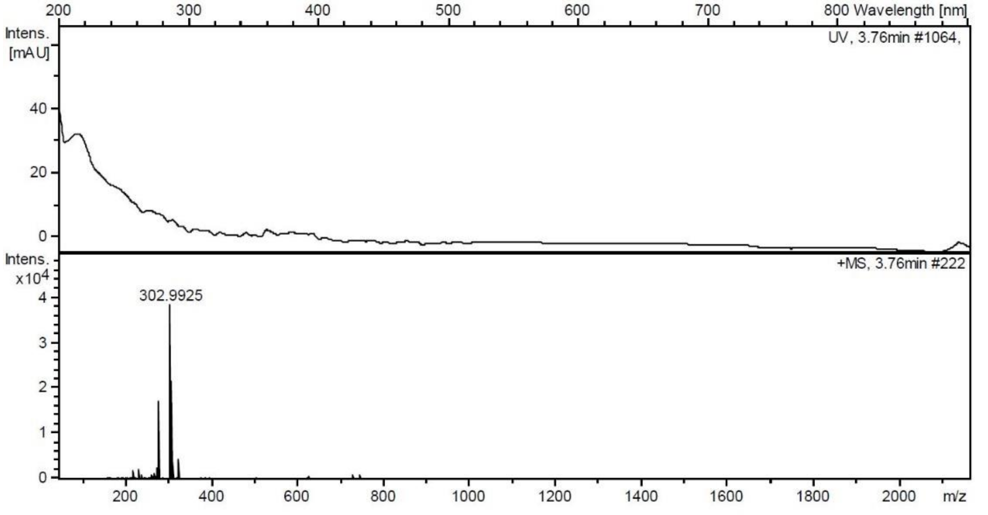

$C_{12}H_{12}O_4$ – Differolide

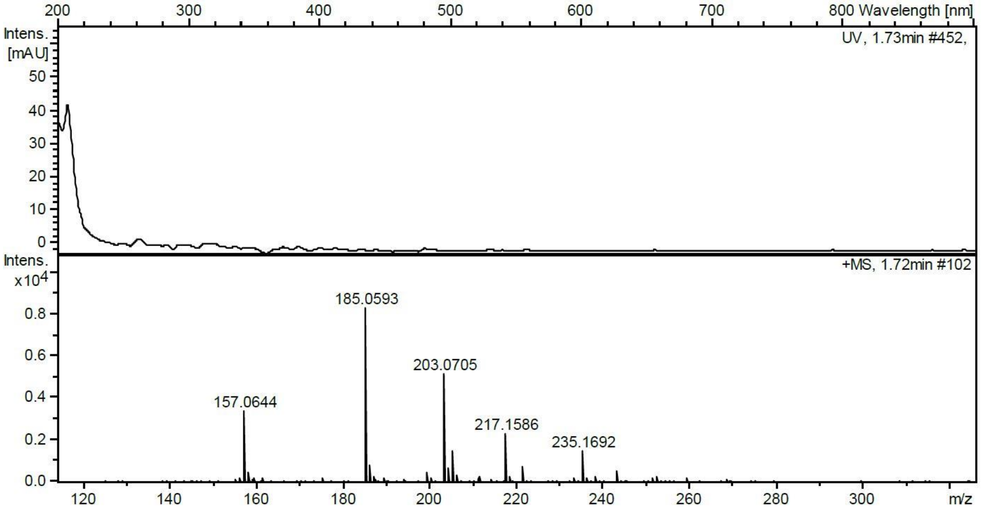

$C_{33}H_{47}Cl_2NO_{13}$* – Putative novel compound

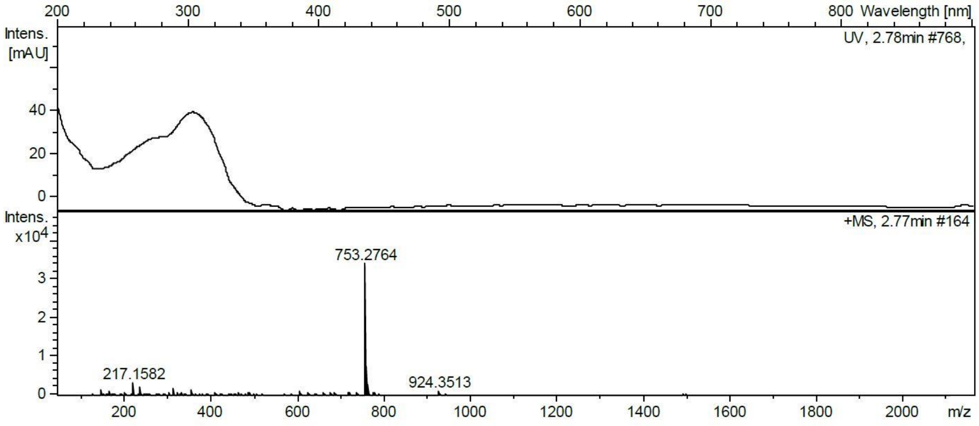

$C_{36}H_{64}N_4O_{10}$* – Putative novel compound

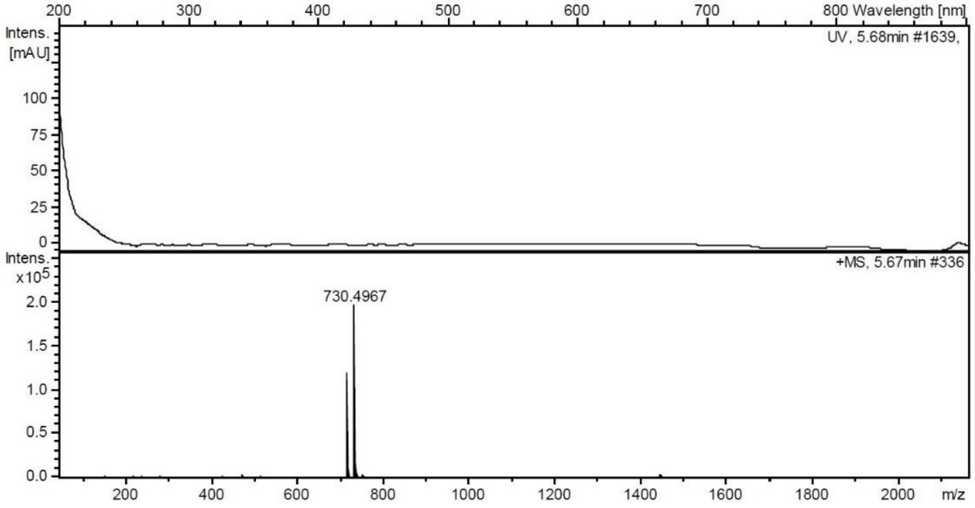

$C_{17}H_{27}Cl_2NO_7$* – Putative novel compound

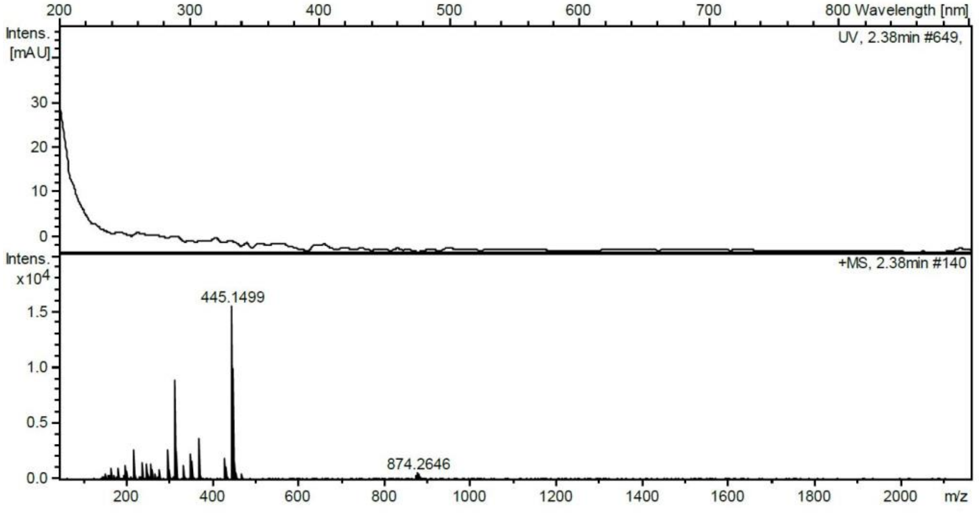

$C_7H_9N_5O_3$* – Putative novel compound

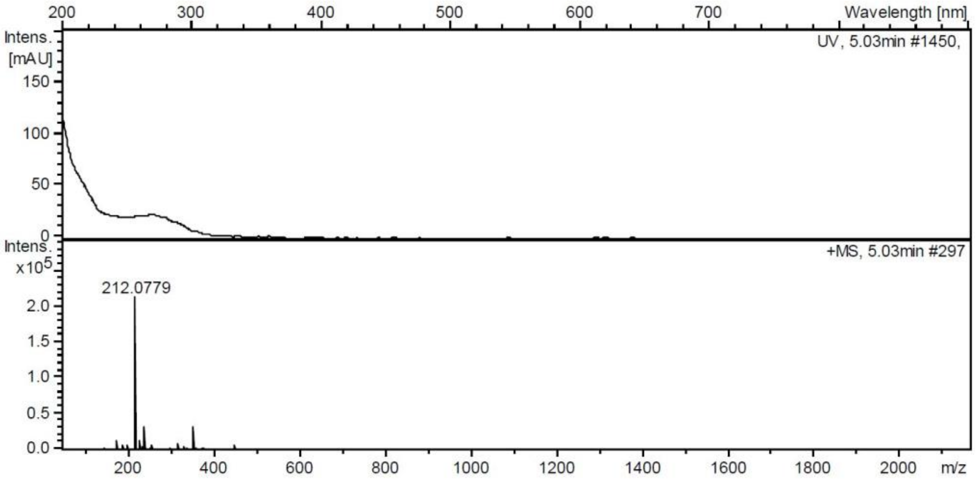

$C_9H_{21}NO_3$* – Putative novel compound

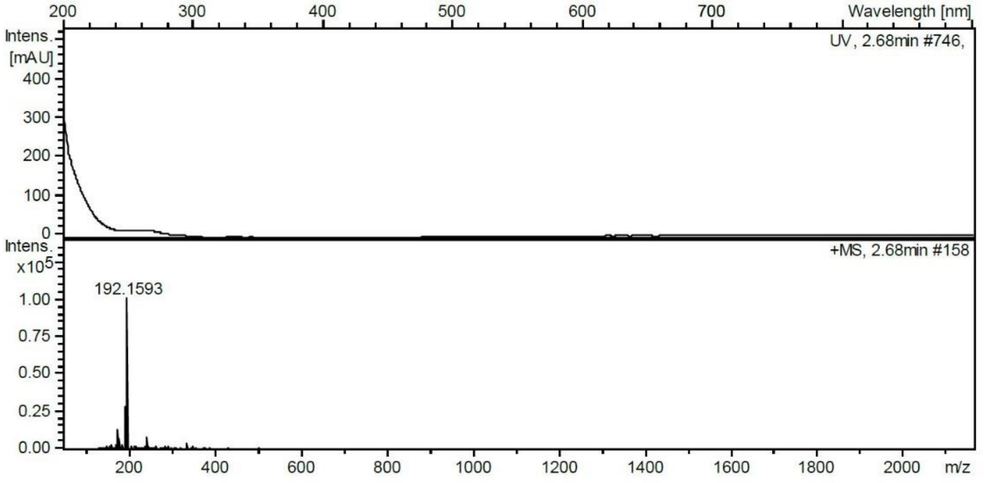

$C_{17}H_{25}N_3O_{13}$* – Putative novel compound

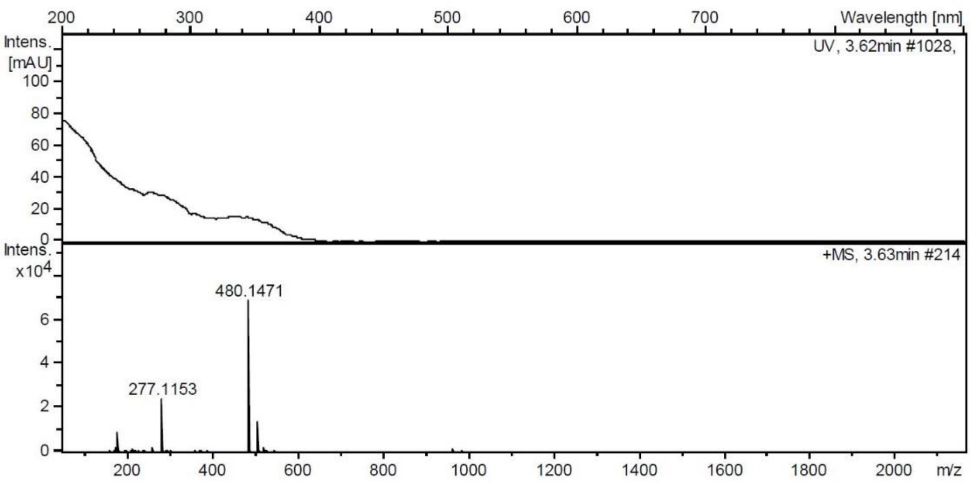

$C_{10}H_9NO_2$* – Putative novel compound

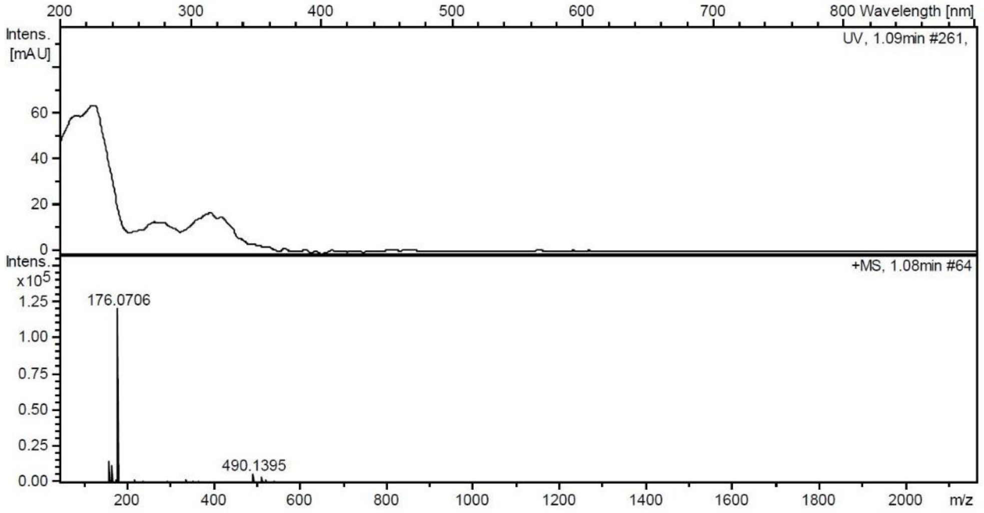

$C_{19}H_{13}N_5O_{12}S$* – Putative novel compound

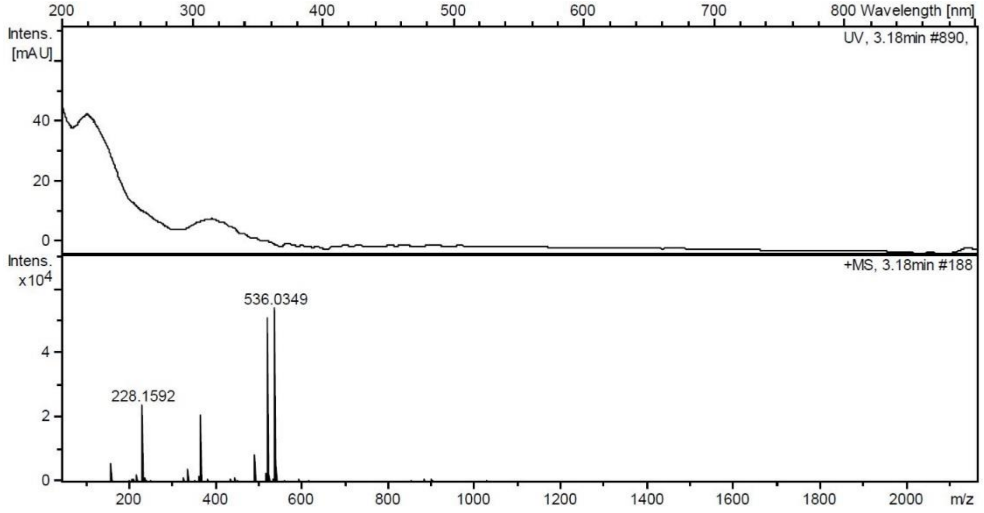

$C_9H_9NO_3$* – Putative novel compound

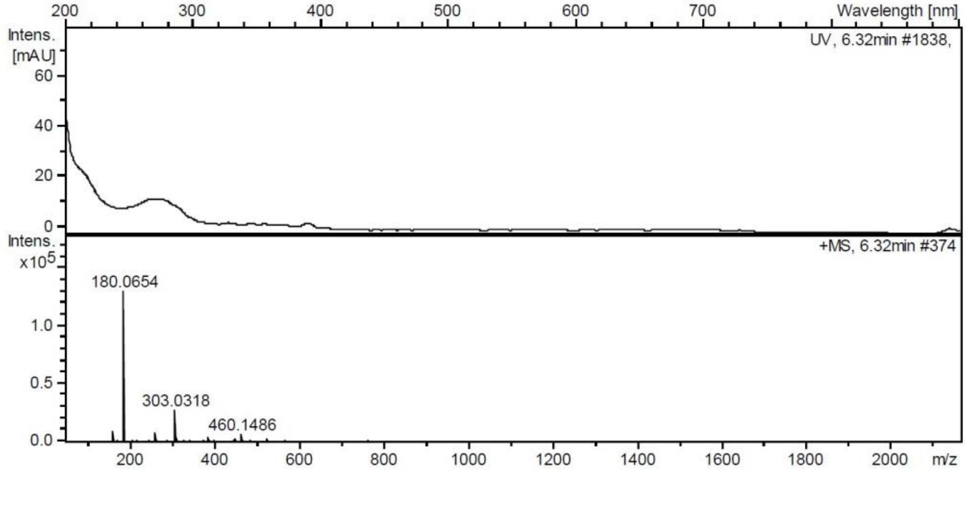

$C_{15}H_{12}O_6S$* – Putative novel compound

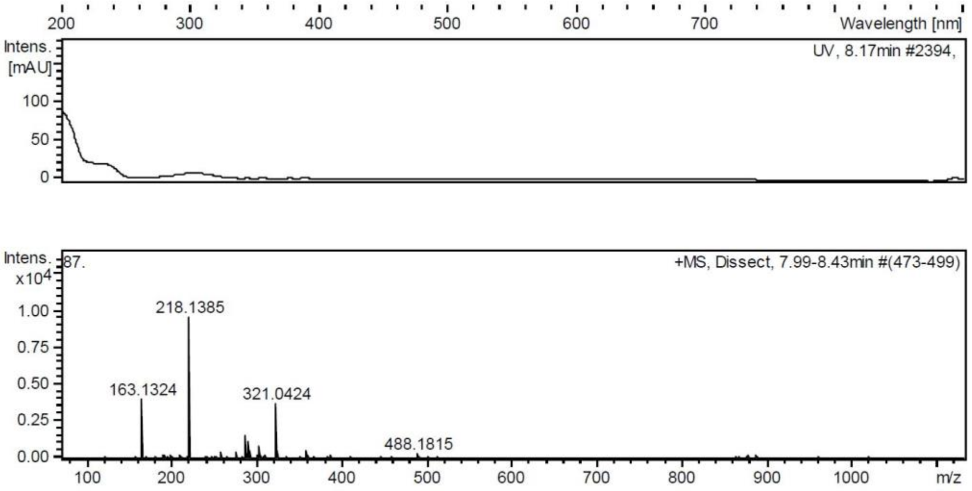

$C_{18}H_{33}N_3O_{12}$* – Putative novel compound

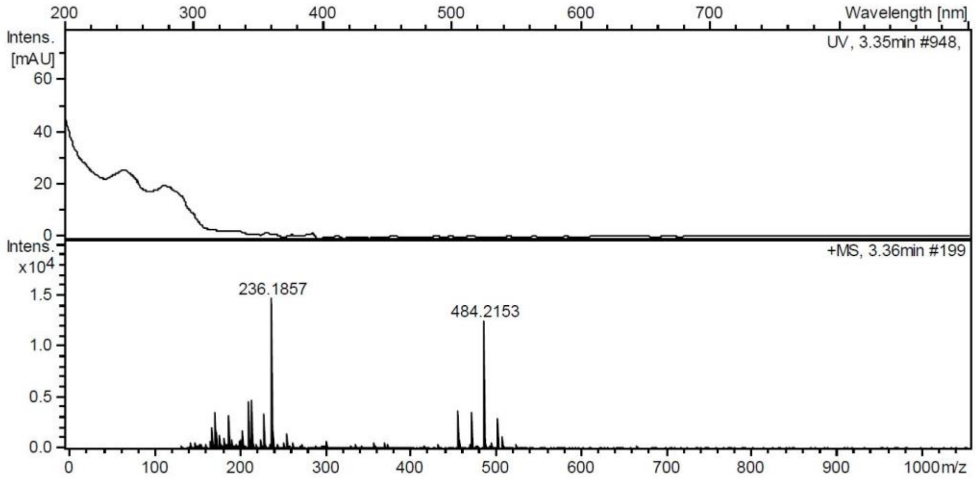

$C_{26}H_{42}O_7$* – Putative novel compound

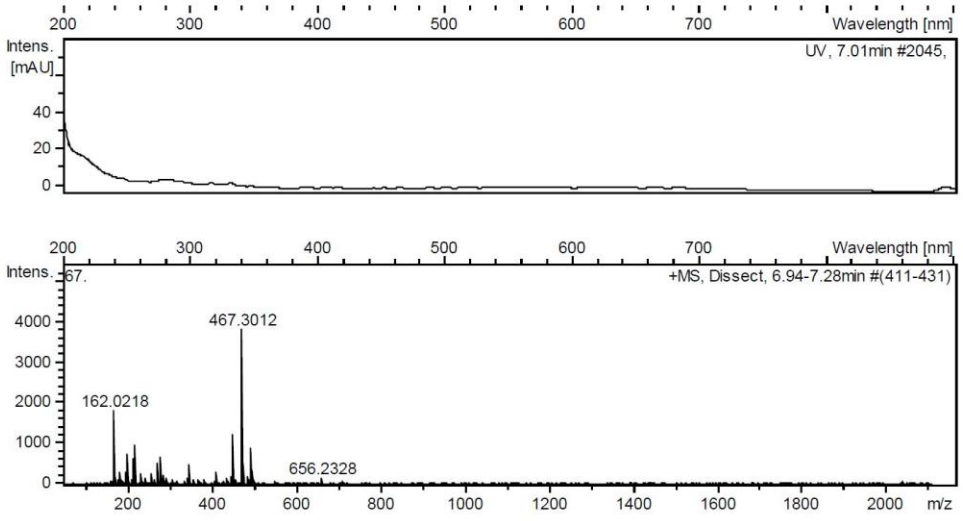

$C_{35}H_{39}N_9O_{11}$* – Putative novel compound

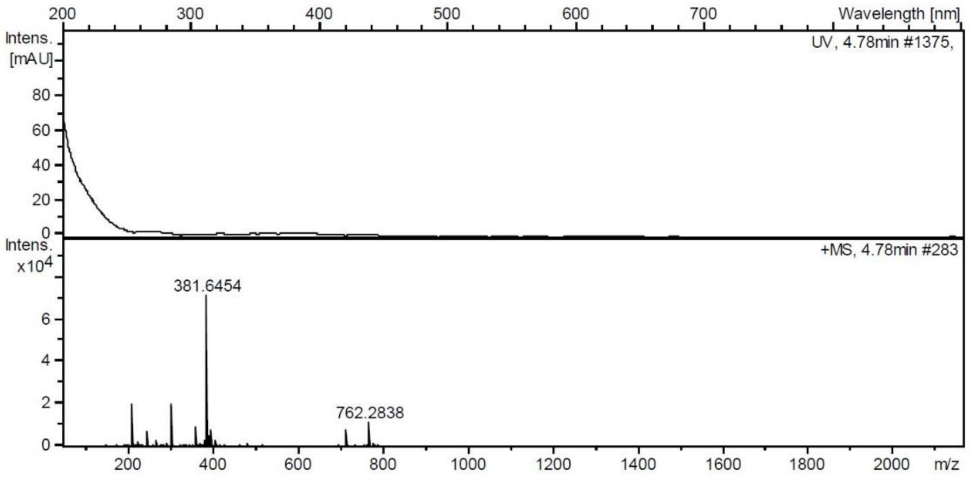

$C_{37}H_{43}N_9O_{11}$* – Putative novel compound

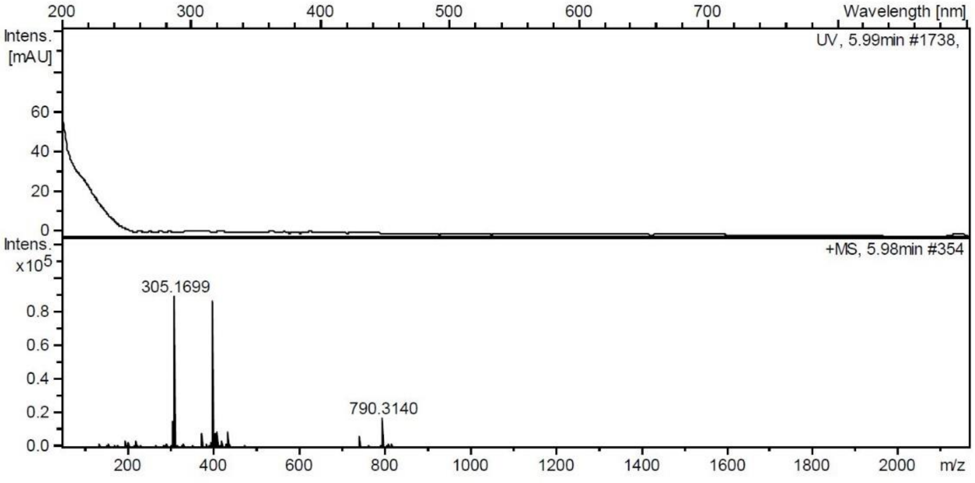

$C_{26}H_{48}N_8O_{14}$* – Putative novel compound

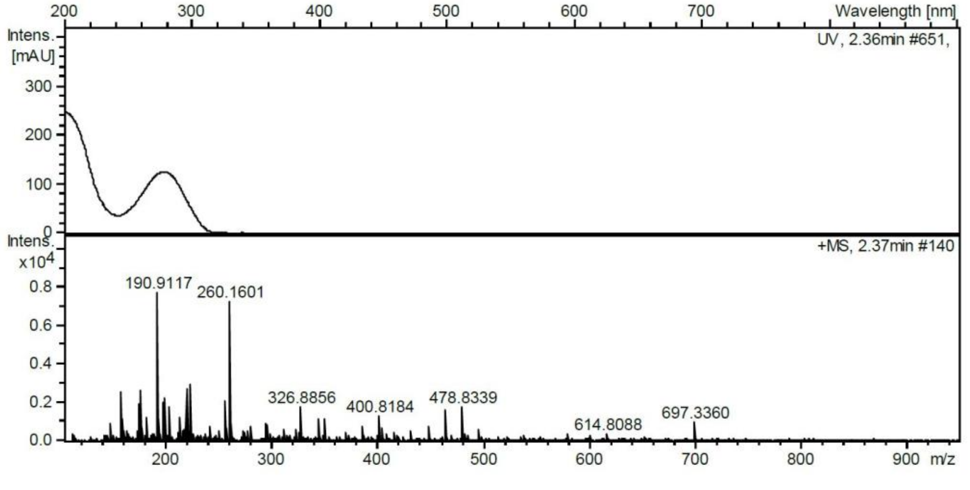

$C_{33}H_{35}N_9O_{11}$* – Putative novel compound

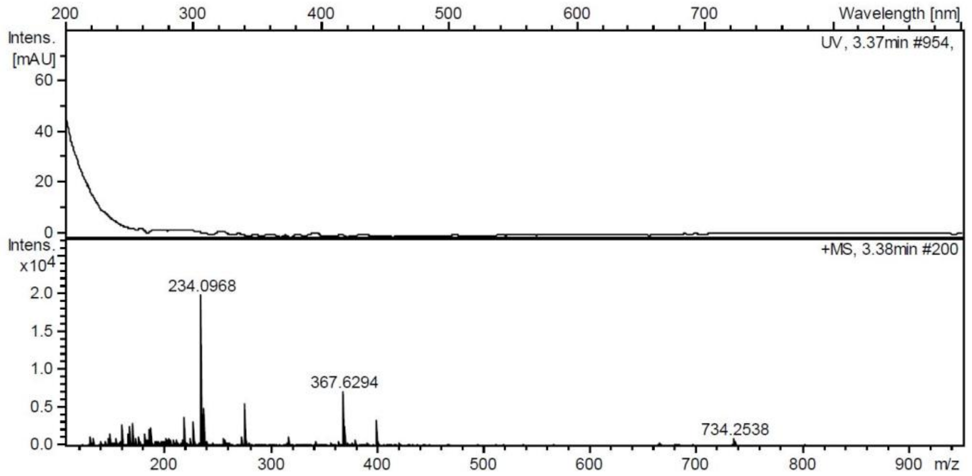

$C_{21}H_{25}FeNO_{15}S_2$* – Putative novel compound

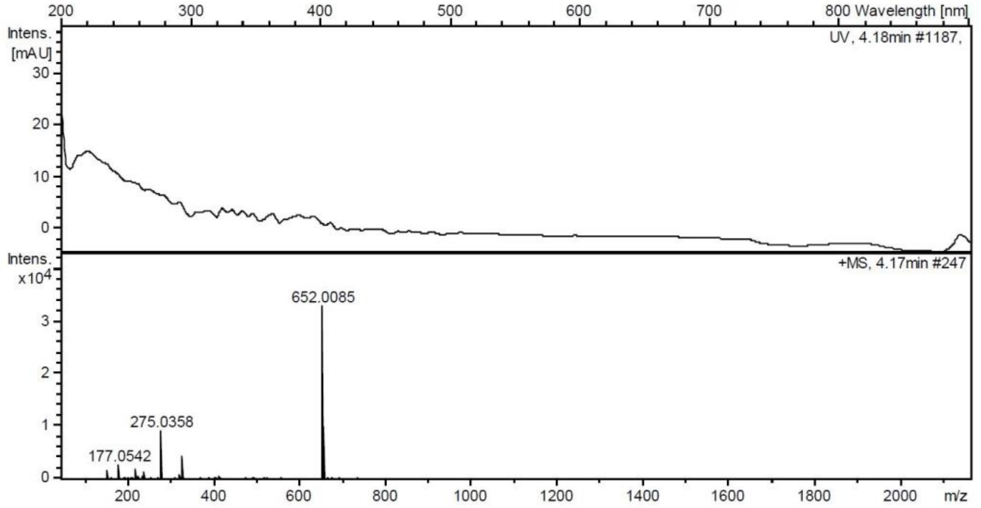

$C_{14}H_{14}O_3$* – Putative novel compound

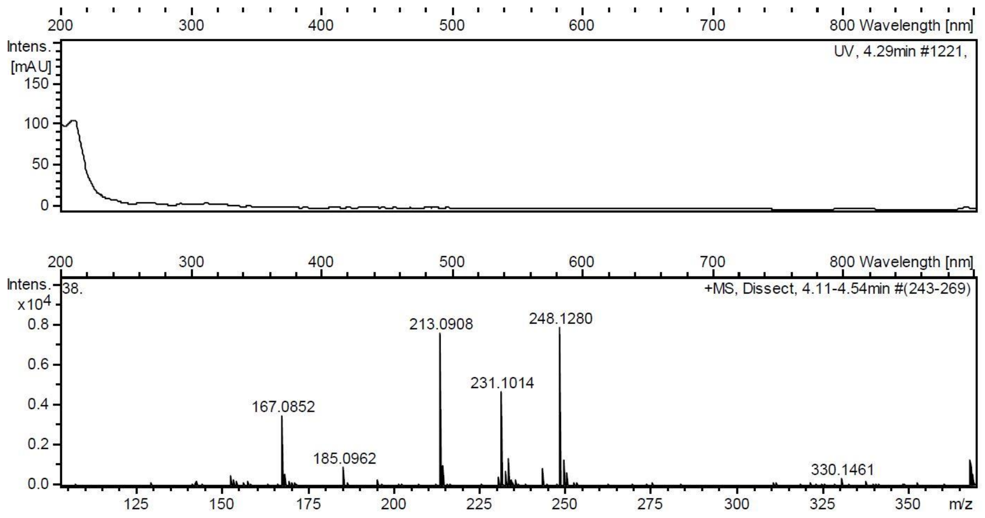

$C_{16}H_{22}O_3$* – Putative novel compound

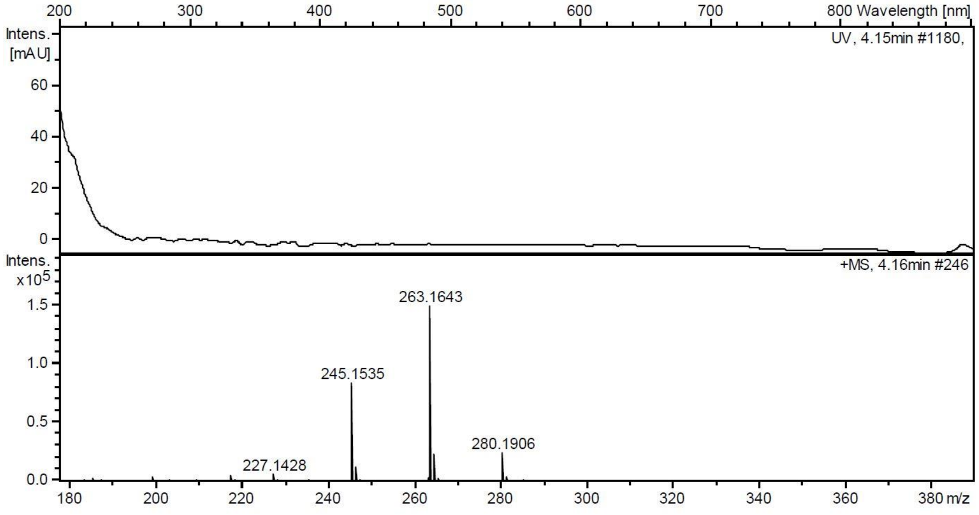

Supplement: Supplementary file 1 [file antibiotics-10-00147-s001.zip › Figure S1.pdf]
