# Supplementary material for: Burkholderia Bacteria Produce Multiple Potentially Novel Molecules that Inhibit Carbapenem-Resistant Gram-Negative Bacterial Pathogens
Source: Antibiotics (Basel). 2021 Feb 2;10(2):147. doi: 10.3390/antibiotics10020147 (PMC7912996; doi:10.3390/antibiotics10020147)

S2 Fig. Antimicrobial activity of semipreparative fractions of *Burkholderia gladioli* R-20794 and *Burkholderia singularis* LMG 28155.

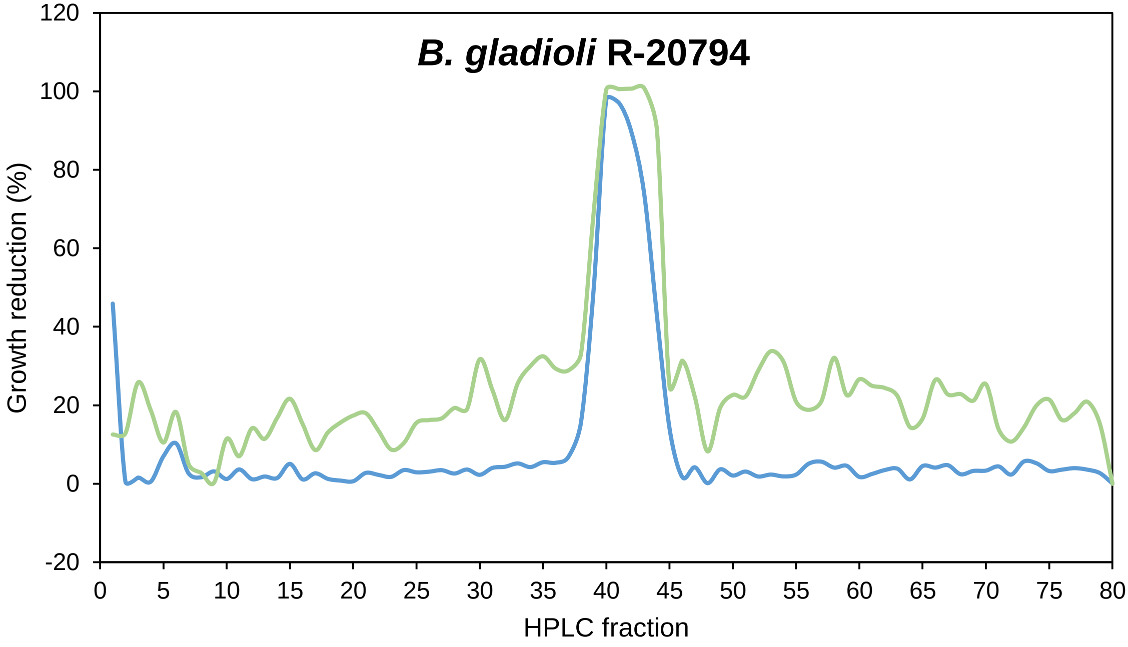

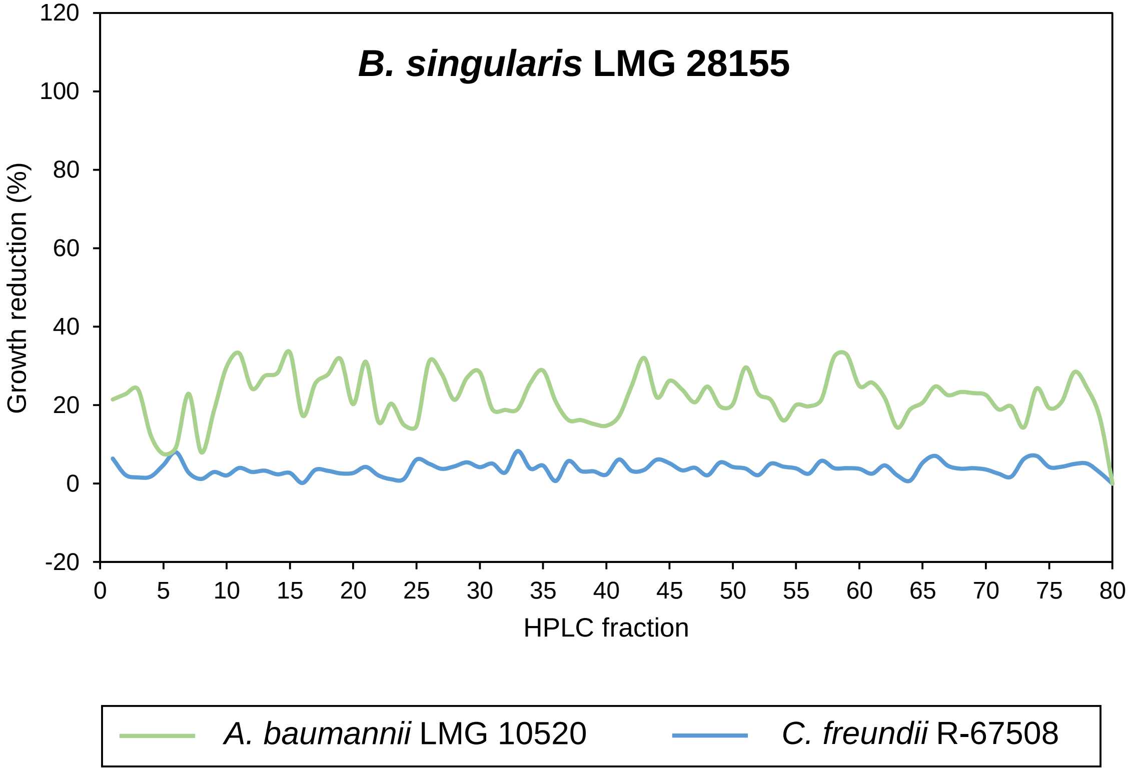

Supplement: Supplementary file 1 [file antibiotics-10-00147-s001.zip › Figure S2.pdf]
