# Supplementary material for: Burkholderia Bacteria Produce Multiple Potentially Novel Molecules that Inhibit Carbapenem-Resistant Gram-Negative Bacterial Pathogens
Source: Antibiotics (Basel). 2021 Feb 2;10(2):147. doi: 10.3390/antibiotics10020147 (PMC7912996; doi:10.3390/antibiotics10020147)
Supplement: Supplementary file 1 [file antibiotics-10-00147-s001.zip › Figure S3.pdf]

S3 Fig. Semipreparative fractionation chromatograms of eight *Burkholderia* isolates. Fractions subsequently analyzed via LC-HRMS are indicated with 'f' and a number, corresponding to the fractions listed in Table 4.

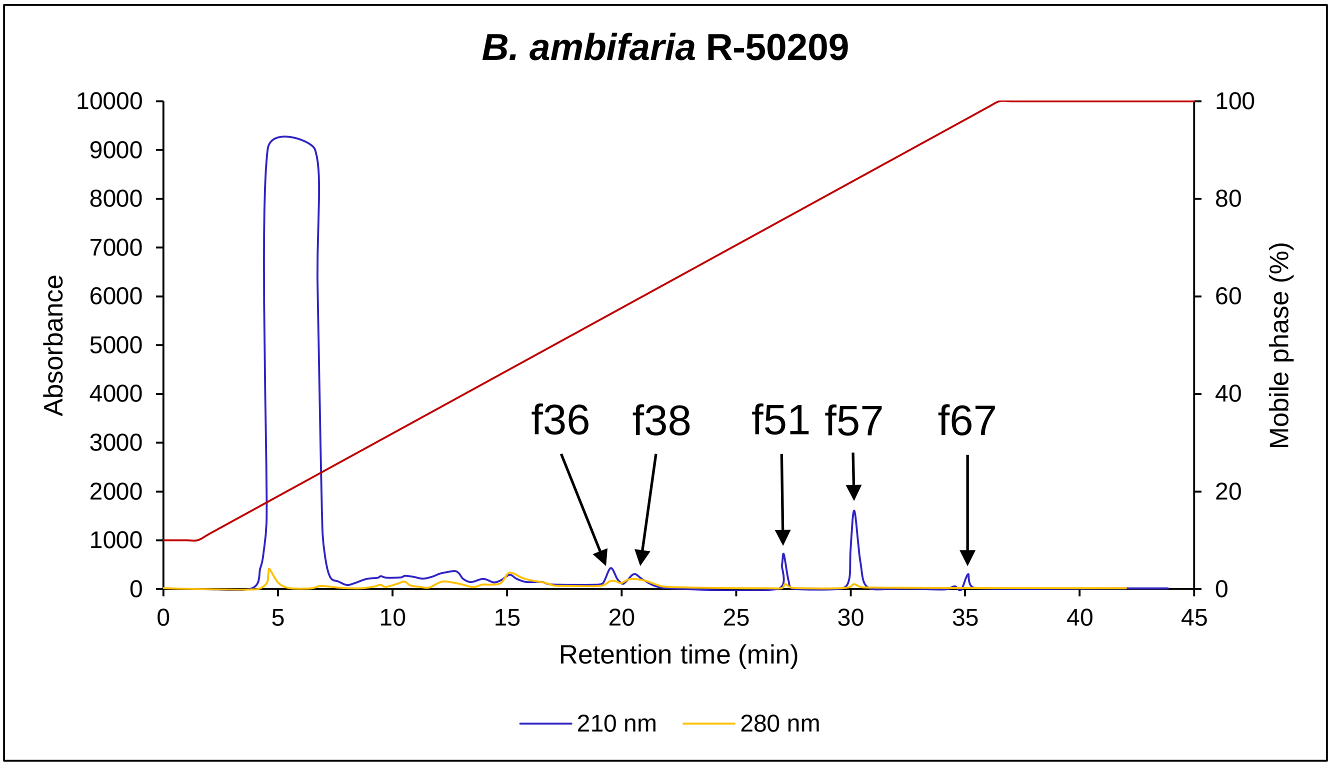

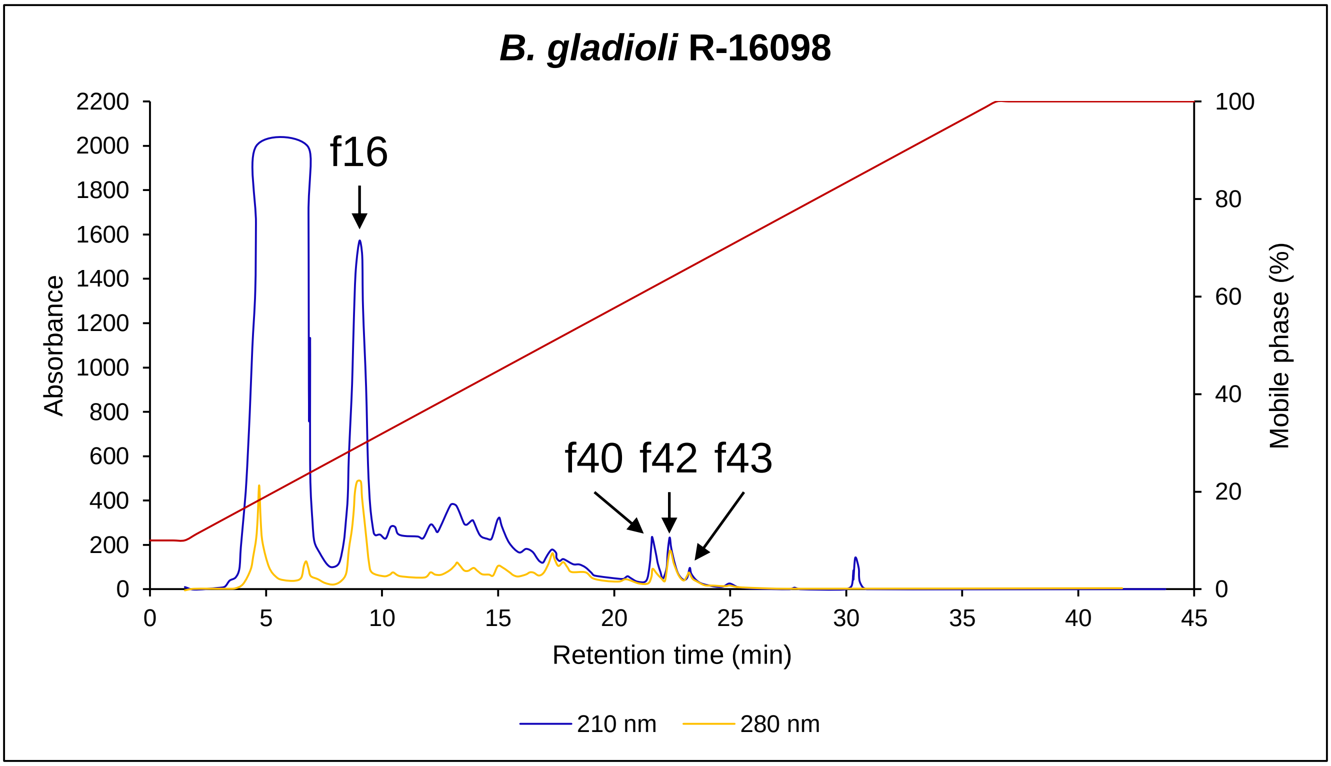

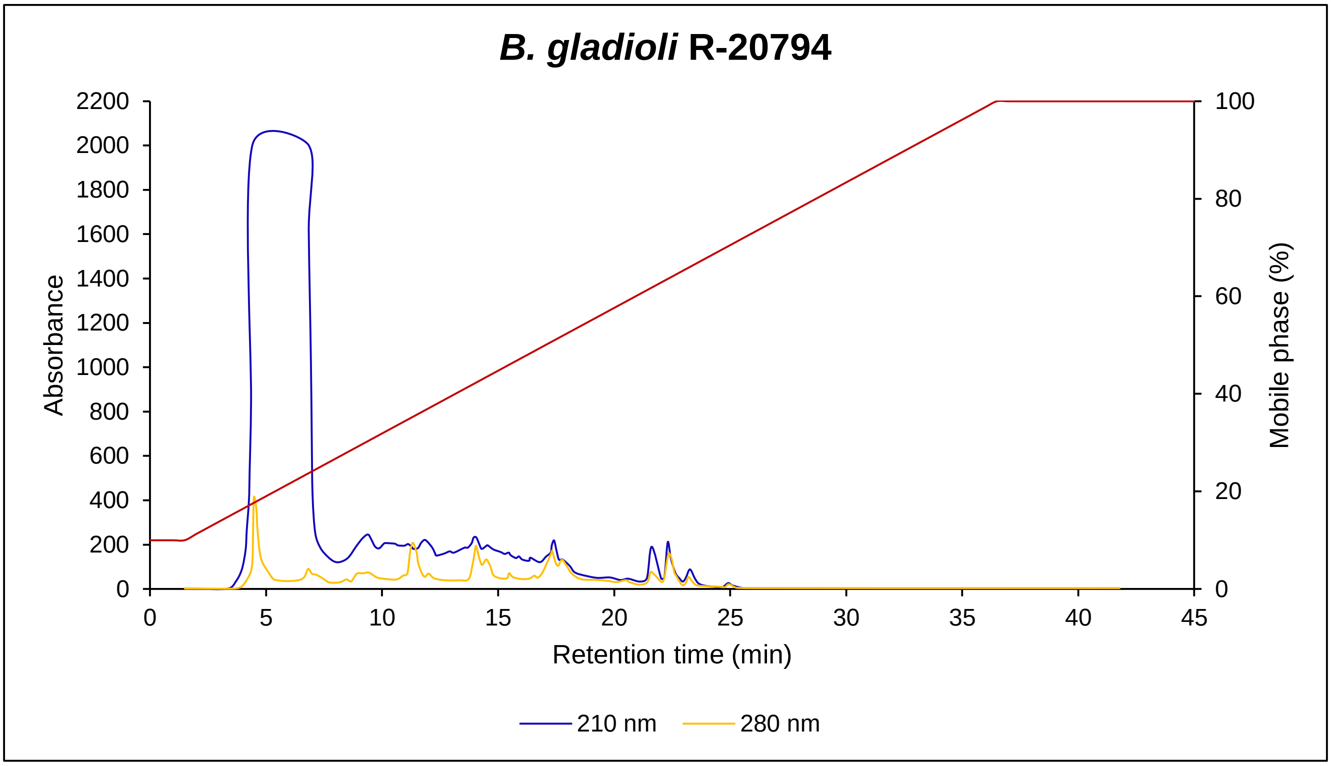

Due to similarity of the profile with strain R-16098, none of the fractions were analyzed via HR-LCMS,

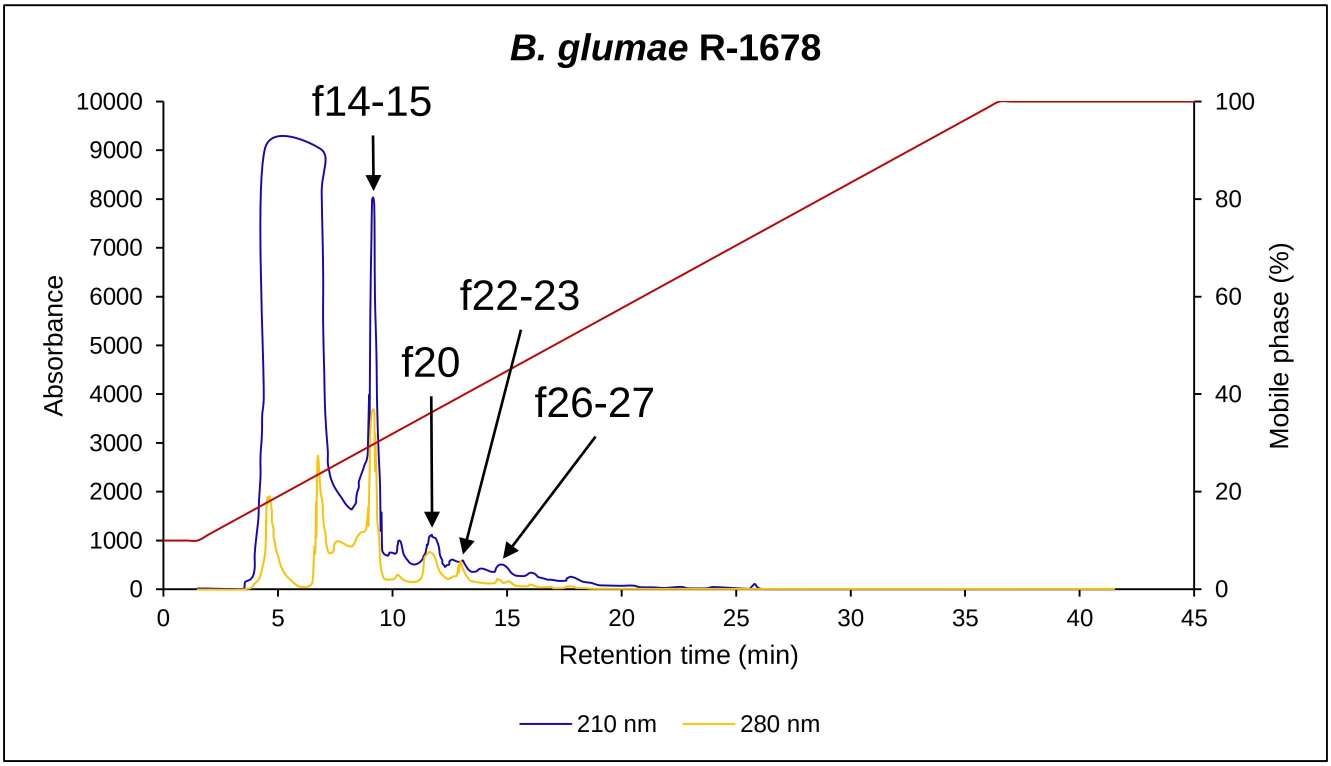

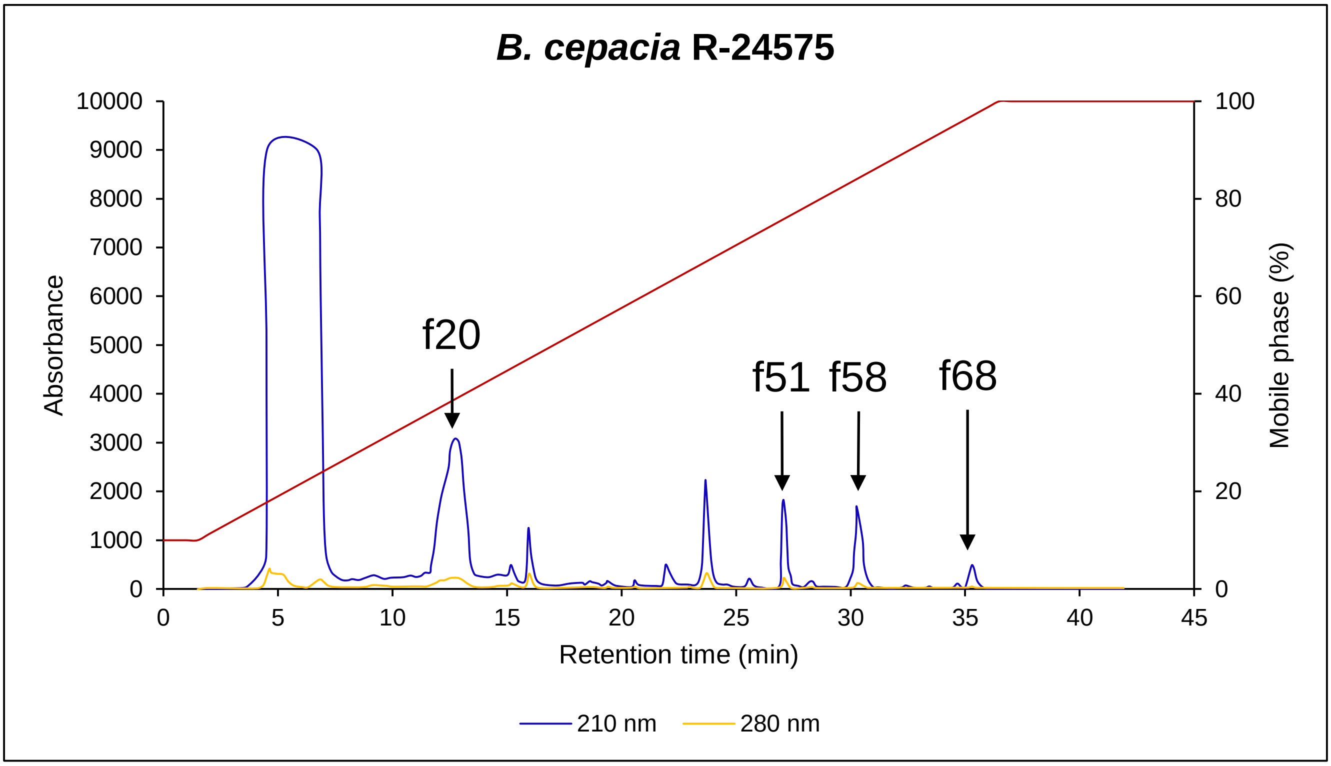

B. cepacia R-24575
Absorbance
Mobile phase (%)
Retention time (min)
f20
f51
f58
f68
0
1000
2000
3000
4000
5000
6000
7000
8000
9000
10000
0
20
40
60
80
100
0 5 10 15 20 25 30 35 40 45
210 nm
280 nm

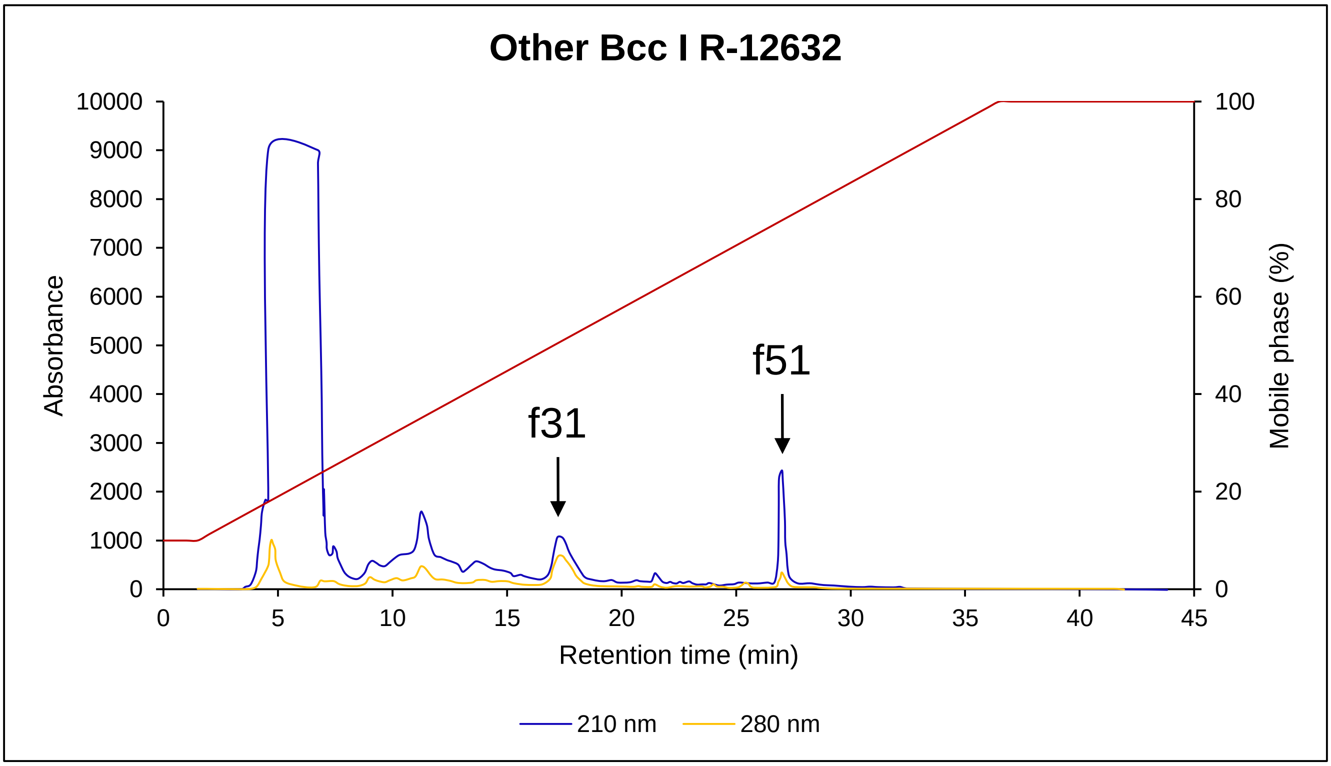

Other Bcc I R-12632
Absorbance
Mobile phase (%)
Retention time (min)
f31
f51
0
1000
2000
3000
4000
5000
6000
7000
8000
9000
10000
0
20
40
60
80
100
0 5 10 15 20 25 30 35 40 45
210 nm
280 nm

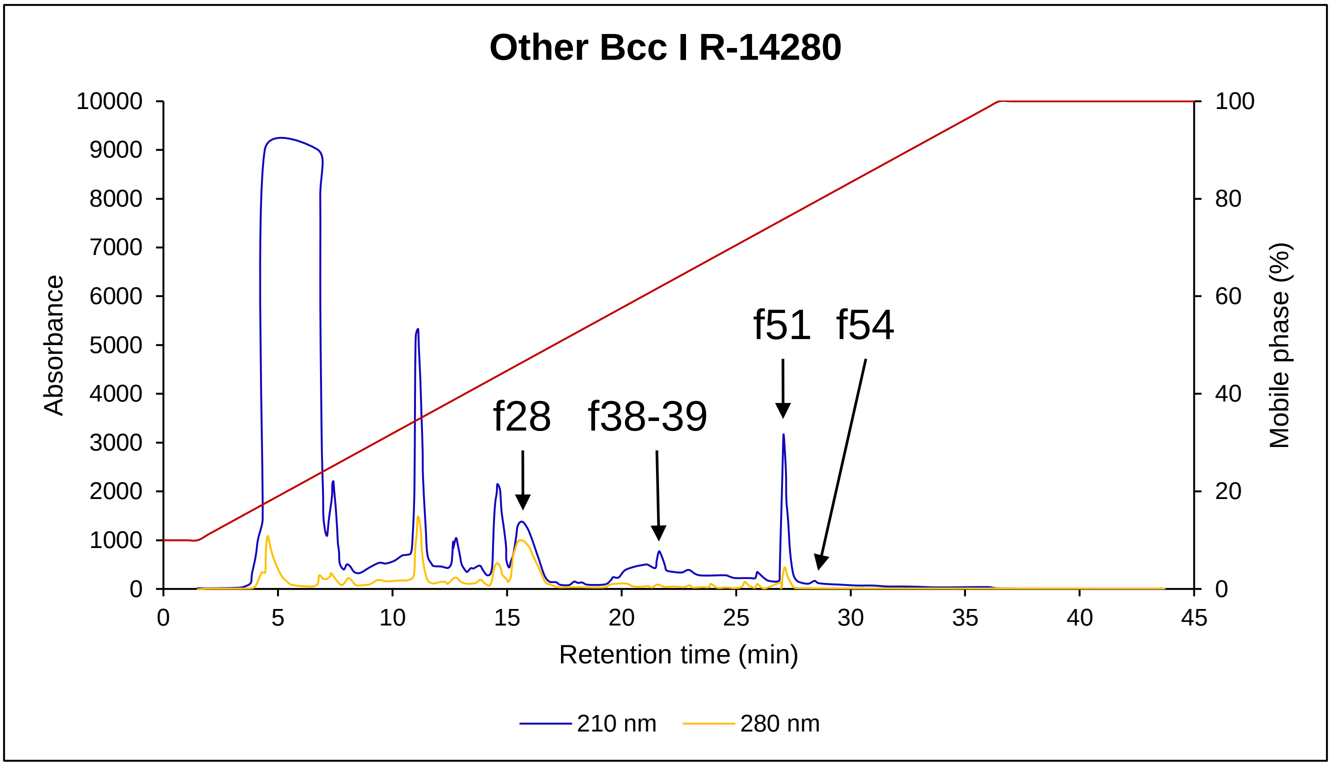

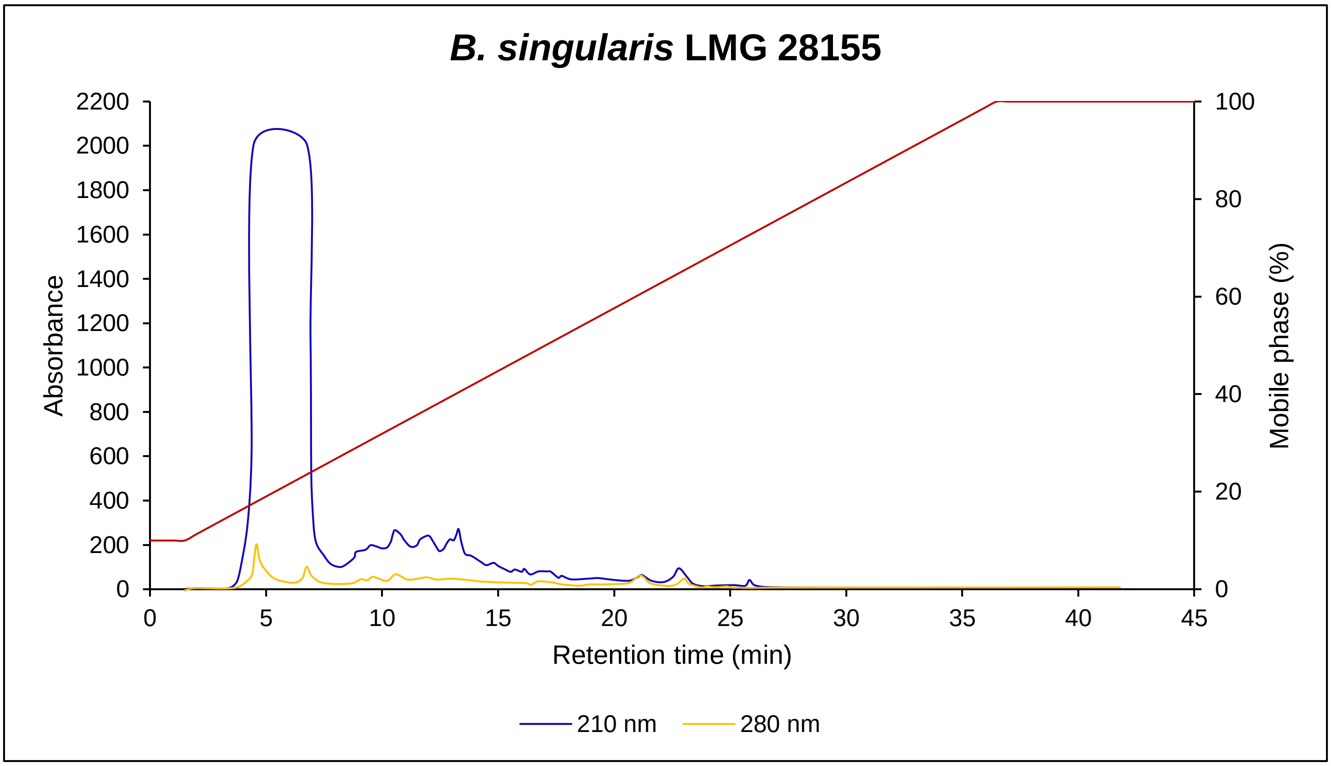

Because none of the fractions showed >40% growth inhibition of at least one of the two pathogens tested, none of the fractions were analyzed through HR-LCMS,
